# Supplementary material for: CRISPR/dCas-mediated counter-silencing: reprogramming dCas proteins into antagonists of xenogeneic silencers
Source: mBio. 2025 May 28;16(7):e00382-25. doi: 10.1128/mbio.00382-25 (PMC12239563; doi:10.1128/mbio.00382-25)
Supplement: Text S1 — Supplemental methods. [file mbio.00382-25-s0001.pdf]

**Text S1. Supplementary information on methods used in this study.**

**Cultivation conditions**

*Cultivation of Escherichia coli DH5α*

*Escherichia coli* DH5α cells were cultivated in Lysogeny Broth (LB) medium (10 g/L tryptone, 5 g/L yeast extract, 10 g/L NaCl) at 37°C for plasmid amplification and storage. Depending on the antibiotic resistance marker of the plasmid, 50 µg/mL kanamycin, 34 µg/mL chloramphenicol, 50 µg/mL apramycin, or 100 µg/mL carbenicillin were added.

*Cultivation of C. glutamicum*

For liquid cultivations, brain heart infusion (BHI) complex medium (Difco, BD, Heidelberg, Germany) (37 g/L BHI) was inoculated with a single *C. glutamicum* colony from a fresh 1.5% (w/v) BHI agar plate and incubated for 8-24 h. If needed, 25 µg/mL kanamycin and/or 10 µg/mL chloramphenicol were added to liquid cultures and plates. BHI pre-cultures were used to inoculate a second overnight pre-culture in CGXII minimal medium (1) that was supplemented with 111 mM glucose and the respective antibiotics. To optimize LacI/$P_{tac}$-derived dCas9 levels, respective cells were pre-cultivated in CGXII medium with 200 µM IPTG. Subsequently, cells from this second pre-culture were used to inoculate a CGXII main culture at a starting OD600 of one. In general, pre-cultures for reporter-based promoter activity assays were performed at 30°C and 900 rpm in 1 ml medium in 2.2 ml square-shaped V-bottom 96-deep well plates (VWR, Radnor, US-PA) covered with a sterile breathable rayon film (VWR, Radnor, US-PA) in a Microtron incubator shaker (Infors, Bottmingen, Switzerland). Other

cultivations were performed in 4.5 ml medium in tubes at 170 rpm or in 25-200 ml in round 0.15-2 L glass shaking flasks with two facing baffles covered with a metal cap at 120 rpm and 30°C. Different amounts of IPTG were added to the cultivation approaches to induce the LacI-dependent $P_{tac}$-derived *dcas9* expression.

*Cultivation of S. venezuelae*

For *S. venezuelae* (NRRL B-65442), strains were grown at 30°C on MS (soy flour-mannitol) agar, MYM (maltose-yeast extract-mannitol) agar, or in liquid MYM medium prepared as described previously (2).

**Recombinant DNA work**

To generate a CRISPR-dCas9 construct for use in *S. venezuelae*, we modified pCRISPR-dCas9 (#AT), which was a gift from Tilmann Weber (Addgene plasmid #125687; http://n2t.net/addgene:125687; RRID:Addgene_125687) (3). To enable use of the thiostrepton-inducible promoter that drives the expression of the dCas9-encoding gene, we first introduced the *tipA* gene (whose product is required for thiostrepton induction) into the plasmid backbone. To minimize leaky expression of the *tipA* gene (and consequently *dcas9*), we introduced a translationally-acting theophylline riboswitch (4) upstream of *tipA*. To eliminate the need for continual plasmid selection, we also introduced the phiC31 *attP-int* locus (5), in place of the pSG5 temperature sensitive origin of replication. Briefly, both the *tipA* and integration elements were successively assembled with the linearized pCRISPR-dCas9 plasmid using Gibson assembly (New England Biolabs, Ipswich, US-MA). To address the fact that this

modification meant that an original NcoI restriction site was no longer unique for guide DNA cloning, the restriction site was changed to a unique AvrII site by removing the NcoI site using the flanking AseI and SnaB1 sites and replacing it with an AvrII site. The sequence and integrity of the CRISPR-dCas9 plasmid (pMC345 (#83)) was finally verified by whole plasmid sequencing (Plasmidsaurus using Oxford Nanopore Technology with custom analysis and annotation (GenBank accession PQ187646, https://www.ncbi.nlm.nih.gov/genbank/)). The two guide RNAs (sgRNA) were individually cloned upstream of the guide RNA scaffold using a protocol for bridging double-stranded DNA with a single-stranded DNA oligo (NEBuilder HiFi DNA assembly).

**Guide RNA design**

The dCas9 and dCas12a-specific guiding sgRNAs and crRNAs were constructed following the designs recently published by Chen and colleagues (6) and by Jiang et al. (7), respectively. For *C. glutamicum*, sgRNAs were designed following a previously established approach, aiming at combining the originally separated (d)Cas9 recruiting tracrRNA and the targeting crRNA (6, 8). sgRNAs were composed of a 20-nucleotide target specific spacer sequence (N-stretch, designed to match its target DNA sequence upstream of a NGG PAM sequence) and the 40-nucleotide sequence for the optimized chimeric dCas9 hairpin (bold letters), followed by the 53-nucleotide *S. pyogenes* terminator (underlined letters).

5´-NNNNNNNNNNNNNNNNNNNN**GTTTAAGAGCTATGCTGGAAACAGCATAGCAAGTTTAAAT**AAGGCTAGTCCGTTATCAACTTGAAAAAGTGGCACCGAGTCGGTGCTTTTTTT-3´

The crRNAs used for the dCas12a approach contained a 19-nucleotide direct repeat dCas12a handle (bold letters) followed by a 20-nucleotide targeting spacer sequence (N-stretch, designed to match its target DNA sequence downstream of a TTTN PAM sequence) and the *rrnB* terminator (T1 and T2 in underlined letters).

5´-**AATTTCTACTGTTGTAGAT**NNNNNNNNNNNNNNNNNNNNCTAGACTCCATTTCTAGACTCCATTTAAATAAAACGAAAGGCTCAGTCGAAAGACTGGGCCTTTCGTTTTATCTGTTGTTTGTCGGTGAACGCTCTCCTGAGTAGGACAAATCCGCCGGGAGCGGATTTGAACGTTGCGAAGCAACGGCCCGGAGGGTGGCGGGCAGGACGCCCGCCATAAACTGCCAGGCATCAAATTAAGCAGAAGGCCATCCTGACGGATGGCCTTTTTGCGTTTCTACAAACTCT-3´

sgRNA expression was driven by constitutive promoters, either by the truncated $P_{tac\Delta}$ (9) or by the $P_{J23119}$ promoter (from Anderson Promoter Collection https://parts.igem.org/Promoters/Catalog/Constitutive). The latter was also used for crRNA expression.

For *S. venezuelae*, two specific-guiding sequences were chosen from the annotated chloramphenicol biosynthetic gene cluster (10) using AGG as the PAM sequence. We focused on sequences within the promoter region of *cmlM* (*sven0925* or *vnz_04460*)

and within the coding strand of *cmU* (*sven0926* or *vnz_04465*), with the latter being a validated Lsr2 binding site (11, 12).

All sgRNA and crRNA spacer sequences are listed in Table S4.

## Fluorescence microscopy

For microscopy, *C. glutamicum* cells were cultivated in 96-deep well plates at 30°C and 900 rpm in CGXII minimal medium supplemented with 111 mM glucose as well as respective antibiotics (25 µg/mL kanamycin and/or 10 µg/mL chloramphenicol) and 200 µM IPTG for *dcas9* expression as required. After 16 h of cultivation, 3 µl cell suspension were dropped onto glass slides coated with a thin 1% (w/v) agarose layer to prevent cell movements. Subsequently, images were acquired with the AxioVision 4.8 software on an Axio Imager M2 microscope equipped with an EC Plan-Neofluar 100x/1.3 Oil Ph3 objective and an AxioCam MRm camera (Zeiss, Oberkochen, Germany). The filter set 46 HE YFP and an exposure time of 1049 ms was used to monitor eYFP fluorescence signals, while Brightfield images were acquired with an illumination time of 18 ms. Shown images resulted from the merged overlay of both channels.

## Microtiter cultivation to monitor growth and fluorescence in reporter assays

Reporter-based analysis of prophage promoter activities was performed in the BioLector I® microtiter system (Beckman Coulter, Brea, US-CA) (13). Unless otherwise indicated, cells were pre-cultivated in CGXII minimal medium supplemented with 111 mM glucose, appropriate antibiotics (25 µg/mL kanamycin and/or 10 µg/mL chloramphenicol) and 200 µM IPTG to maximize dCas9 levels. Subsequently, 750 µl CGXII main cultures with a starting OD600 of one were incubated in 48 FlowerPlates® (Beckman Coulter, Brea, US-CA) covered with a sterile breathable rayon film (VWR, Radnor, US-PA) at 30°C, 1200 rpm and 85% humidity. Online monitoring of growth and fluorescence was performed in 15 min intervals. Here, biomass production was recorded as backscattered light intensity of sent light with a wavelength of 620 nm (signal gain factor 20). Venus fluorescence signals were measured with an excitation wavelength of 508 nm and emission wavelength of 532 nm (signal gain factor 60). Arbitrary units (a.u.) of backscatter-normalized reporter outputs (specific fluorescence) were calculated by dividing the Venus signal by the backscatter signal per time point (13). To determine the effect of specific sgRNAs, fold changes were computed based on the specific Venus fluorescence ratios from cells expressing the sgRNA versus those that lack any sgRNA encoding sequence. For direct comparison of signals that differ in their dynamics, integrals over 24 h of cultivation of backscatter-normalized Venus reporter outputs were calculated with the area under curve function from the software GraphPad prism 8.00 (GraphPad Software, La Jolla, US-CA). The same software was applied to plot all graphs and to determine significance levels (two-tailed, unpaired *t*-

tests). Unless otherwise indicated, all graphs represent means of biological triplicates (n=3) and error bars the corresponding standard deviations.

## Transcriptome analysis

*Cultivation conditions*

After a first BHI pre-culture in tubes (4.5 ml) in the presence of 10 µg/mL chloramphenicol, cells were incubated overnight in 30 ml CGXII minimal medium supplemented with 111 mM glucose, 10 µg/mL chloramphenicol and 200 µM IPTG in 150 ml shaking flasks. For RNA sequencing, the main cultures were inoculated with a starting OD600 of one in 200 ml CGXII medium containing the same supplements as in the second pre-culture. After 7 h of cultivation, 25 ml of the cultures were mixed with ice and cells from the mid-exponential phase were harvested by centrifugation (15 min, 11,325 x g and 4°C). Cell pellets were snap-frozen in liquid nitrogen and stored at -80°C until use. To determine the relative amount of circular CGP3 in both strains, cultivations were continued, and samples were harvested after 24 h.

*RNA isolation and Illumina sequencing*

Total RNA was isolated using the Monarch Total RNA Miniprep Kit (New England Biolabs, Ipswich, US-MA). Library preparation, rRNA depletion, and Illumina NovaSeq 2x150 bp sequencing with 10 M read pairs was performed by Genewiz (Leipzig, Germany). The sequencing results, including quality control of the reads and trimming of adapter sequences, low-quality reads (0.05) and ambiguous nucleotides were

analyzed with the software CLC genomics workbench v.24 (Qiagen, Hilden, Germany). The *C. glutamicum* ATCC 13032 genome (NCBI reference sequence NC_003450.3) either combined with the sequences of plasmids pEC-XC99E-*lacI*-$P_{tac}$-*dcas9*--$P_{tac\Delta}$-sgRNA-CS3 (#2) or pEC-XC99E-*lacI*-$P_{tac}$-*dcas9*--$P_{tac\Delta}$-sgRNA-CS1 (#5) served as reference for read mapping. The ´RNA-Seq Analysis´ tool of CLC was used to calculate the transcripts per million (TPM) values (chosen parameters: mismatch cost 2; insertion cost 3; deletion cost 3; length fraction 0.9; similarity fraction 0.9; maximum number of hits for a read: 10; strand specificity: both; library type: bulk; expression level: ignore broken pairs). The ´Differential Expression in Two Groups´ function of CLC was applied for identifying significantly differentially expressed genes (FDR-*P*-values<0.05 and |$\log_2$ fold change values|>1) in clones expressing $P_{cg1974_}$sgRNA-CS3 (#2) compared to the combined $P_{cg1974_}$sgRNA-CS1 reference cells (#5) (full data set can be found in Table S5) and for generating the read coverage profiles (data aggregation: 100 bp) and the volcano plots.

*Off-target identification*

The Cas-OFFinder online tool (http://www.rgenome.net/cas-offinder/) from Bae and colleagues (14) was used to identify putative off-targets of sgRNA-CS1 and -CS3. Analyses were performed with the settings SpCas9 from *Streptococcus pyogenes* (5'-NGG-3'). Strict conditions forbidding any sgRNA-DNA binding mismatches or ´DNA/RNA bulges´ and with strongly relaxed conditions by allowing up to five mismatches and up to two ´DNA/RNA bulges´ were applied. The *C. glutamicum* ATCC 13032 genome (NCBI reference sequence BA000036.3, equivalent to NC_003450.3)

served as reference. All identified non-overlapping off-target positions are given in Table S6. Putative off-targets were assigned to a gene locus if they were located in the gene (gene) or within 1000 nucleotides of its upstream sequence (intergenic region/promoter). Here, a putative binding region was designated as promoter if the off-target position was located within the 300-nucleotide region upstream of the gene locus.

**Quantitative PCR (qPCR)**

For the quantification of the relative amount of circular CGP3 prophage DNA, *C. glutamicum* wild-type cells co-expressing *dcas9* and sgRNA-CS3 (#2) or –CS1 (#5) were cultivated as described in section "Transcriptome analysis" in Text S1. After 24 h of cultivation, cells were harvested from 5 ml cultures by centrifugation (15 min, 11,325 x g and 4°C) and shock-frozen in liquid nitrogen. Cell pellets were stored at -80°C until use. The genomic DNA was isolated using the NucleoSpin microbial DNA Kit (Macherey Nagel, Dueren, Germany) and was quantified with a nanophotometer (Implen, Munich, Germany). qPCR was performed in biological triplicates and technical duplicates with the Luna® Universal qPCR Master Mix (New England Biolabs, Ipswich, US-MA) with 50 ng total DNA in a qTOWER 2.2 (Analytic Jena, Jena, Germany) following the manufacturer´s instructions. Oligonucleotides B040/B041 were used to detect the circularized CGP3 prophage DNA element, while oligonucleotides B042/B043 amplified a fragment from the *ddh* (NCgl2528) reference gene (Table S3C). Both PCR products

were 150 bp long. Data evaluation was done with the qPCR software qPCR 3.1 (Analytik Jena, Jena, Germany) and the $C_T$ value-based $2^{-\Delta\Delta CT}$ method from Livak and Schmittgen (15). Here, geometric averages of biological triplicates and technical duplicates of $C_T$ values were used to determine the $2^{-\Delta\Delta CT}$ values, shown as dots. The error bars represent the possible ranges, determined by $2^{-\Delta\Delta CT+SD(\Delta\Delta CT)}$ as minimal range and $2^{-\Delta\Delta CT\text{-}SD(\Delta\Delta CT)}$ as maximum. The error values SD($\Delta C_T$) for sgRNA-CS3 and –CS1 expressing samples were calculated with the following equation:

$$SD(\Delta CT) = \sqrt{SD\ circ.CGP3^2 + SD\ ddh^2} \quad (1)$$

SD($\Delta\Delta C_T$) values were calculated with the following equation:

$$SD(\Delta\Delta CT) = \sqrt{SDCS3\ \Delta CT^2 + SDCS1\ \Delta CT^2} \quad (2)$$

## Analysis of *Streptomyces* metabolite extracts

Metabolites were extracted from 5 mL cultures of *S. venezuelae* strains grown in maltose-yeast extract-malt extract (MYM; 4 g/L maltose, 4 g/L yeast extract, 10 g/L malt extract) liquid medium for three days. Induction of the *dcas9* gene was conducted using 2 mM theophylline and 0-50 µg/mL thiostrepton (same conditions were used for *dcas9* lacking E339 control). Culture broths were separated by centrifugation and subjected to Diaion HP-20 resin (3% w/v) for 8 h. The resin was washed twice using 5 mL distilled water, separated from the supernatant using filter paper, and dried overnight.

Metabolites were eluted from the dried resin using 0.5 ml methanol, followed by solvent removal under reduced pressure using a vacuum concentrator (Thermo Scientific, Ashville, US-NC). The resulting pellet was resuspended in 50 µl of HPLC grade water:methanol (1:1). After the removal of particulates by centrifugation, the concentrated extracts were subjected to LC-MS analyses under UV and ion detection.

Analytical HPLC was performed using an Agilent 1290 Infinity LC System with a Zorbax Eclipse XDB C18 column (100 mm × 2.1 mm × 3.5 µm), coupled to an LTQ Orbitrap XL MS system (Thermo Scientific, Waltham, US-MA). Metabolites were separated at a flow rate of 0.4 ml/min using a 7 min solvent gradient, from 90% solvent A (water with 0.1% formic acid) and 10% solvent B (100% acetonitrile) to 50% A and 50% B, a 2.5 min gradient to 5% A and 95% B, a 3 min isocratic flow under 5% A and 95% B. HR-ESI-MS analysis was conducted under negative scan mode over a mass range of 100 to 1000 Da, followed by Fourier transform mass spectrometry (FTMS) ion extraction for $C_{11}H_{12}Cl_2N_2O_5$ chloramphenicol, $[M-H]^-$ *m/z* 321.00.

**REFERENCES**


1. Keilhauer C, Eggeling L, Sahm H. 1993. Isoleucine synthesis in *Corynebacterium glutamicum*: molecular analysis of the *ilvB-ilvN-ilvC* operon. J Bacteriol 175:5595-5603.
2. Kieser T, Bibb M, Buttner M, Chater K, Hopwood D. 2000. Practical *streptomyces* genetics, vol 59. John Innes Foundation, Norwich, UK.
3. Tong Y, Charusanti P, Zhang L, Weber T, Lee SY. 2015. CRISPR-Cas9 Based Engineering of Actinomycetal Genomes. ACS Synth Biol 4:1020-9.

4. Rudolph MM, Vockenhuber MP, Suess B. 2013. Synthetic riboswitches for the conditional control of gene expression in *Streptomyces coelicolor*. Microbiol (Reading) 159:1416-1422.
5. Bierman M, Logan R, O'Brien K, Seno ET, Rao RN, Schoner BE. 1992. Plasmid cloning vectors for the conjugal transfer of DNA from *Escherichia coli* to *Streptomyces* spp. Gene 116:43-9.
6. Chen B, Gilbert LA, Cimini BA, Schnitzbauer J, Zhang W, Li GW, Park J, Blackburn EH, Weissman JS, Qi LS, Huang B. 2013. Dynamic imaging of genomic loci in living human cells by an optimized CRISPR/Cas system. Cell 155:1479-91.
7. Jiang Y, Qian F, Yang J, Liu Y, Dong F, Xu C, Sun B, Chen B, Xu X, Li Y, Wang R, Yang S. 2017. CRISPR-Cpf1 assisted genome editing of *Corynebacterium glutamicum*. Nat Commun 8:15179.
8. Jinek M, Chylinski K, Fonfara I, Hauer M, Doudna JA, Charpentier E. 2012. A programmable dual-RNA-guided DNA endonuclease in adaptive bacterial immunity. Science 337:816-21.
9. Gilbert LA, Horlbeck MA, Adamson B, Villalta JE, Chen Y, Whitehead EH, Guimaraes C, Panning B, Ploegh HL, Bassik MC, Qi LS, Kampmann M, Weissman JS. 2014. Genome-scale CRISPR-mediated control of gene repression and activation. Cell 159:647-661.
10. Fernández-Martínez LT, Borsetto C, Gomez-Escribano JP, Bibb MJ, Al-Bassam MM, Chandra G, Bibb MJ. 2014. New insights into chloramphenicol biosynthesis in *Streptomyces venezuelae* ATCC 10712. Antimicrob Agents Chemother 58:7441-50.
11. Gehrke EJ, Zhang X, Pimentel-Elardo SM, Johnson AR, Rees CA, Jones SE, Hindra, Gehrke SS, Turvey S, Boursalie S, Hill JE, Carlson EE, Nodwell JR, Elliot MA. 2019. Silencing cryptic specialized metabolism in *Streptomyces* by the nucleoid-associated protein Lsr2. Elife 8.
12. Zhang X, Andres SN, Elliot MA. 2021. Interplay between nucleoid-associated proteins and transcription factors in controlling specialized metabolism in *Streptomyces*. mBio 12:e0107721.
13. Kensy F, Zang E, Faulhammer C, Tan RK, Büchs J. 2009. Validation of a high-throughput fermentation system based on online monitoring of biomass and fluorescence in continuously shaken microtiter plates. Microb Cell Fact 8:31.
14. Bae S, Park J, Kim J-S. 2014. Cas-OFFinder: a fast and versatile algorithm that searches for potential off-target sites of Cas9 RNA-guided endonucleases. Bioinformatics 30:1473-1475.
15. Livak KJ, Schmittgen TD. 2001. Analysis of relative gene expression data using real-time quantitative PCR and the $2^{-\Delta\Delta CT}$ method. Methods 25:402-408.
