# Supplementary material for: CRISPR/dCas-mediated counter-silencing: reprogramming dCas proteins into antagonists of xenogeneic silencers
Source: mBio. 2025 May 28;16(7):e00382-25. doi: 10.1128/mbio.00382-25 (PMC12239563; doi:10.1128/mbio.00382-25)
Supplement: Supplemental Material — Tables S1 to S4 and Figures S1 to S6. [file mbio.00382-25-s0002.pdf]

SUPPLEMENTARY INFORMATION TO

CRISPR/dCas-mediated counter-silencing: reprogramming dCas proteins into antagonists of xenogeneic silencers

Johanna Wiechert[a], Biel Badia Roigé[a], Doris Dohmen-Olma[a], Hindra[b,c], Xiafei Zhang[b,c], Roberto G. Stella[a], Marie A. Elliot[b,c] and Julia Frunzke[a#]

[a]Institute of Bio- and Geoscience – IBG-1: Biotechnology, Forschungszentrum Juelich, Juelich, Germany

[b]Department of Biology and Michael G. DeGroote Institute for Infectious Disease Research, McMaster University, Hamilton, Ontario, Canada

[c]Michael G. DeGroote Institute for Infectious Disease Research, McMaster University, Hamilton, Ontario, Canada

#Adress correspondence to Julia Frunzke, j.frunzke@fz-juelich.de

TEXT SUPPLEMENTARY MATERIAL

Text S1. Supplementary information on methods used in this study. (Separate file)

TABLES SUPPLEMENTARY MATERIAL

Table S1. Strains used in this study.

Table S2. Plasmids used in this study.

Table S3A. Oligonucleotides used for plasmid constructions (see Table S2).

Table S3B. Oligonucleotides used for sequencing and colony PCR (see Table S2).

Table S3C. Oligonucleotides used for qPCR.

Table S4. Overview of guide RNA spacer sequences.

Table S5. RNA-seq - Differential gene expression (cg1974_sgRNA-CS3 clone 1 (A) and 2 (B)). (Excel file)

Table S6. Off-targets of cg1974_sgRNA-CS1 (A) and –CS3 (B). (Excel file)

FIGURES SUPPLEMENTARY MATERIAL

Figure S1. Correlation of CRISPRcosi efficiencies at prophage promoter P_{cg1974} (A) and P_{cg2014} (B) and *dcas9* expression levels.

Figure S2. The crRNA binding positions determine the dCas12a-mediated CRISPRcosi efficiency.

Figure S3. Supplementary information on Figure 5: dCas9 and dCas12a both mediate efficient CRISPRcosi.

Figure S4. Supplementary information on Figure 6: CRISPRcosi counteracts CgpS-mediated silencing at different prophage promoters.

Figure S5. Supplementary data on Figure 7: Investigation of potential off-target effects of CRISPRcosi.

Figure S6. Supplementary data on Figure 9: Impact of CRISPR counter silencing and interference in antibiotic (chloramphenicol) production by *Streptomyces venezuelae*.

Table S1. Strains used in this study.

The published strain names are given in brackets after the reference in case of deviations.

Strain	Relevant characteristics	Reference
Escherichia coli DH5α	F$^-$ Φ80*lacZ*ΔM15 Δ(*lacZYA-argF*) U169 *recA1 endA1 hsdR17*(r_k^-, m_k^+) *phoA supE44 thi-1 gyrA96 relA1* λ$^-$, strain used for cloning procedures	Invitrogen
E. coli ET12567 (pUZ8002)	F$^-$ *dam13*::Tn9 *dcm6 hsdM hsdR recF143 zjj201*::Tn10 *galK2 galT22 ara14 lacY1 xyl5 leuB6 thi1 tonA31 rpsL136 hisG4 tsx78 mtli glnV44*, pUZ8002 (helper plasmid)<br><br>non-methylating *E. coli* strain used for conjugation of *Streptomyces*	(1)
Corynebacterium glutamicum ATCC 13032	Biotin-auxotrophic *C. glutamicum* wild-type	(2)
C. glutamicum ATCC 13032::P_{cg1974}-*eyfp*	Derivative of ATCC 13032 containing the prophage reporter P_{cg1974}-*eyfp* integrated into the intergenic region of cg1121-cg1122	(3) (ATCC 13032::P_{lys}-*eyfp*)
Δphage	Derivative of ATCC 13032 with deletion of prophages CGP1 (cg1507-cg1524), CGP2 (cg1746-cg1752), and CGP3 (cg1890-cg2071)	(4) (MB001)
Δphage::P_{cgpS}-*cgpS*	Derivative of Δphage (MB001) with re-integrated *cgpS* together with its native promoter P_{cgpS} in the intergenic region of cg1199-cg1201	(5)
Streptomyces venezuelae NRRL B-65442	*S. venezuelae* wild-type	(6)
E327A	*S. venezuelae* Δ*lsr2*	(7)

E339	*S. venezuelae* wild-type carrying the plasmid pIJ6902, in which the apramycin resistance gene has been replaced with a hygromycin resistance gene	This work

Table S2. Plasmids used in this study.

All constructed plasmids were based on the pJC1, pSM22 or the pEC-XC99E vector systems providing a kanamycin (*kan*^{R}), carbenicillin (*amp*^{R}) or chloramphenicol resistance (*cm*^{R}), respectively. pJC1- (pCG1 replication origin) and pEC-XC99E- (pGA1 replication origin) based plasmids were transferred into *C. glutamicum* cells. If needed, pJC1- and pEC-XC99E-derivatives were co-transferred as a double-plasmid system. pSM22-based plasmids were designed as intermediate constructs to facilitate cloning of pJC1 and pEC-XC99E derivatives. All plasmids used for *S. venezuelae* were based on the pCRISPR-dCas9 plasmid which was a gift from Tilmann Weber (Addgene plasmid #125687; http://n2t.net/addgene:125687 RRID:Addgene_125687) (8). We assigned an ID (indicated by #) to all plasmids and categorized them into two different groups. Group 1, labelled with letters, contains plasmids used from other sources (#a-#d) and from this work (#A-#AV) that were not used in experiments but were used as helper vectors for plasmid construction. Group 2 contains plasmids from this work and other sources (in case of differences, the published plasmid names are given in brackets after the reference) that were used to characterize the principle of CRISPRcosi in experiments. These plasmids were numbered (#1-#85) according to their first mention in the results section. Oligonucleotide pairs used in PCRs for insert amplification (Table S3A) are given as numbers in the column "Construction of plasmids used in this work". DNA templates are listed in brackets behind the oligonucleotides. Chromosomal *C. glutamicum* ATCC 13032 DNA was isolated as

noted previously (9) and served as PCR template for insert amplification if indicated. Plasmid backbones including the restriction enzymes used for linearization are given (*). Oligonucleotides used for colony PCR (C) and Sequencing (S) are listed (Table S3B). Information about promoter regions bound by CgpS were based on previous work from Pfeifer et al. (10).

Plasmid ID	Plasmid	Construction of plasmids used in this work	Relevant characteristics	Oligonucleotides used for colony PCR (C) and sequencing (S)	Source or reference
#a	pEC-XC99E		*cm*^{R}, *E. coli*/*C. glutamicum* shuttle vector (*cat*$_I$, *lacI*^{q}, P$_{trc}$, *rrnB* (T1 and T2), *oriV*$_{Ec}$, *per*, *repA* (pGA1 *ori*$_{Cg}$)		(11)
#b	pPBEx2		*kan*^{R}, *E. coli*/*C. glutamicum* shuttle vector (P$_{tacI}$; *lacI*^{q}; *ori*$_{Cg}$ from pBL1, *ori*$_{Ec}$ ColE1 from pUC18)		(12)
#c	#44251 (addgene)		*amp*^{R}, *E. coli* vector (*ori*$_{Ec}$ ColE1 from pUC19, contains the P$_{J23119\ (SpeI)}$-guide RNA (bacteria) sequence)		(13)
#d	pCLTON1		*kan*^{R}, *E. coli*/*C. glutamicum* shuttle vector (*B. subtilis*		(14)

			derived P_{gap}-*tetR*__P_{tet} expression system, $oriV_{Cg}$, $oriV_{Ec}$)		
#A	pSM22	Three consecutive site-directed mutagenesis (SDM) rounds (1st: OSM35/OSM36; 2nd: OSM37/OSM38; 3rd: OSM47/OSM48 on plasmid #c	*amp*^{R}, #44251 (#c) derivative containing the $P_{J23119\ (SpeI)}$-promoter followed by an optimized sgRNA hairpin sequence, based on (13)		Gift from Susana Matamouros
#B	pSM24	Gibson assembly: OSM13/OSM14 into (#d)*PstI, followed by SDM with OSM39/OSM40	*kan*^{R}, pCLTON1^{TS} (RepA^{P47S}) (#d) derivative containing the *dcas9* gene		Gift from Susana Matamouros
#C	pEC-XC99E-*dcas9*-sgRNA_hairpin-T_{Sp}	Gibson assembly: R69/R70 (#A) and R67/R68 (#B) into #a *NdeI *SalI	*cm*^{R}, pEC-XC99E (#a) derivative containing a promoter-less *dcas9* gene and, in divergent orientation and separated by a linker sequence containing the XmaI and SpeI sites, the sequence encoding a structurally optimized sgRNA hairpin followed by a *Streptococcus*	C: R71/R72<br>S: R71, R72, R73, R74, R75, R76, R77, R78	This work

			pyogenes-derived terminator (13) (the term sgRNA hairpin includes the hairpin and the terminator in the following)		
#D	pEC-XC99E-*lacI*-P_{tac}-*dcas9*--$P_{tac\Delta}$-SpeI_site-sgRNA_hairpin-T_{Sp}	Gibson assembly R91/R92 (#b) and R93/R94 (#b) into #C *SpeI *XmaI	*cm^R*, pEC-XC99E-*dcas9*-sgRNA_hairpin-T_{Sp} (#C) derivative with an inserted *$lacI^q$*-P_{tac} sequence upstream of *dcas9* and a truncated, constitutive $P_{tac\Delta}$ promoter upstream of the sgRNA hairpin sequence, separated by a SpeI site	C: R95/R96<br>S: R169, R171	This work
#E	pSM22-P_{J32119}-P_{cg1974}_sgRNA-CS3	Gibson assembly 864/865 (#A *SpeI), circularized	*amp^R*, pSM22 derivative with an inserted P_{cg1974}_sgRNA-CS3 spacer encoding sequence at the SpeI site between the P_{J23119} promoter and the sgRNA hairpin	S: 890	This work
#F	pSM22-P_{J32119}-P_{cg1974}_sgRNA-25	Gibson assembly 1711/1712 (#A *SpeI), circularized	*amp^R*, pSM22 derivative with an inserted P_{cg1974}_sgRNA-25 spacer encoding sequence at the SpeI site between the P_{J23119} promoter	S: 890	This work

			and the sgRNA hairpin		
#G	pSM22-P_{J32119}-P_{cg1974}_sgRNA-26	Gibson assembly 1713/1714 (#A *SpeI), circularized	*amp*^{R}, pSM22 derivative with an inserted P_{cg1974}_sgRNA-26 spacer encoding sequence at the SpeI site between the P_{J23119} promoter and the sgRNA hairpin	S: 890	This work
#H	pSM22-P_{J32119}-P_{cg1974}_sgRNA-28	Gibson assembly 1715/1716 (#A *SpeI), circularized	*amp*^{R}, pSM22 derivative with an inserted P_{cg1974}_sgRNA-28 spacer encoding sequence at the SpeI site between the P_{J23119} promoter and the sgRNA hairpin	S: 890	This work
#I	pSM22-P_{J32119}-P_{cg1974}_sgRNA-29	Gibson assembly 1717/1718 (#A *SpeI), circularized	*amp*^{R}, pSM22 derivative with an inserted P_{cg1974}_sgRNA-29 spacer encoding sequence at the SpeI site between the P_{J23119} promoter and the sgRNA hairpin	S: 890	This work
#J	pSM22-P_{J32119}-P_{cg1974}_sgRNA-CS1	Gibson assembly 1719/1720 (#A *SpeI), circularized	*amp*^{R}, pSM22 derivative with an inserted P_{cg1974}_sgRNA-CS1 spacer encoding sequence at the	S: 890	This work

			SpeI site between the P_{J23119} promoter and the sgRNA hairpin		
#K	pSM22-P_{J32119}-P_{cg1974}_sgRNA-33	Gibson assembly 1721/1722 (#A *SpeI), circularized	*amp*^{R}, pSM22 derivative with an inserted P_{cg1974}_sgRNA-33 spacer encoding sequence at the SpeI site between the P_{J23119} promoter and the sgRNA hairpin	S: 890	This work
#L	pSM22-P_{J32119}-P_{cg1974}_sgRNA-34	Gibson assembly 1723/1724 (#A *SpeI), circularized	*amp*^{R}, pSM22 derivative with an inserted P_{cg1974}_sgRNA-34 spacer encoding sequence at the SpeI site between the P_{J23119} promoter and the sgRNA hairpin	S: 890	This work
#M	pSM22-P_{J32119}-P_{cg1974}_sgRNA-35	Gibson assembly 1725/1726 (#A *SpeI), circularized	*amp*^{R}, pSM22 derivative with an inserted P_{cg1974}_sgRNA-35 spacer encoding sequence at the SpeI site between the P_{J23119} promoter and the sgRNA hairpin	S: 890	This work
#N	pSM22-P_{J32119}-P_{cg1974}_sgRNA-36	Gibson assembly 1727/1728 (#A	*amp*^{R}, pSM22 derivative with an inserted P_{cg1974}_sgRNA-36	S: 890	This work

		*SpeI), circularized	spacer encoding sequence at the SpeI site between the P_{J23119} promoter and the sgRNA hairpin		
#O	pSM22-P_{J32119}-P_{cg1974}_sgRNA-37	Gibson assembly 1729/1730 (#A *SpeI), circularized	*amp*^{R}, pSM22 derivative with an inserted P_{cg1974}_sgRNA-37 spacer encoding sequence at the SpeI site between the P_{J23119} promoter and the sgRNA hairpin	S: 890	This work
#P	pSM22-P_{J32119}-P_{cg1974}_sgRNA-38	Gibson assembly 1731/1732 (#A *SpeI), circularized	*amp*^{R}, pSM22 derivative with an inserted P_{cg1974}_sgRNA-38 spacer encoding sequence at the SpeI site between the P_{J23119} promoter and the sgRNA hairpin	S: 890	This work
#Q	pSM22-P_{J32119}-P_{cg1974}_sgRNA-39	Gibson assembly 1733/1734 (#A *SpeI), circularized	*amp*^{R}, pSM22 derivative with an inserted P_{cg1974}_sgRNA-39 spacer encoding sequence at the SpeI site between the P_{J23119} promoter and the sgRNA hairpin	S: 890	This work

#R	pSM22-P_{J32119}-P_{cg1974}_sgRNA-40	Gibson assembly 1735/1736 (#A *SpeI), circularized	*amp*^{R}, pSM22 derivative with an inserted P_{cg1974}_sgRNA-40 spacer encoding sequence at the SpeI site between the P_{J23119} promoter and the sgRNA hairpin	S: 890	This work
#S	pSM22-P_{J32119}-P_{cg2014}_sgRNA-22	Gibson assembly 1659/1660 (#A *SpeI), circularized	*amp*^{R}, pSM22 derivative with an inserted P_{cg2014}_sgRNA-22 spacer encoding sequence at the SpeI site between the P_{J23119} promoter and the sgRNA hairpin	S: 890	This work
#T	pSM22-P_{J32119}-P_{cg2014}_sgRNA-24	Gibson assembly 1661/1662 (#A *SpeI), circularized	*amp*^{R}, pSM22 derivative with an inserted P_{cg2014}_sgRNA-24 spacer encoding sequence at the SpeI site between the P_{J23119} promoter and the sgRNA hairpin	S: 890	This work
#U	pSM22-P_{J32119}-P_{cg2014}_sgRNA-25	Gibson assembly 1663/1664 (#A *SpeI), circularized	*amp*^{R}, pSM22 derivative with an inserted P_{cg2014}_sgRNA-25 spacer encoding sequence at the SpeI site between the P_{J23119} promoter	S: 890	This work

			and the sgRNA hairpin		
#V	pSM22-P_{J32119}-P_{cg2014}_sgRNA-26	Gibson assembly 1665/1666 (#A *SpeI), circularized	*amp*^{R}, pSM22 derivative with an inserted P_{cg2014}_sgRNA-26 spacer encoding sequence at the SpeI site between the P_{J23119} promoter and the sgRNA hairpin	S: 890	This work
#W	pSM22-P_{J32119}-P_{cg2014}_sgRNA-27	Gibson assembly 1667/1668 (#A *SpeI), circularized	*amp*^{R}, pSM22 derivative with an inserted P_{cg2014}_sgRNA-27 spacer encoding sequence at the SpeI site between the P_{J23119} promoter and the sgRNA hairpin	S: 890	This work
#X	pSM22-P_{J32119}-P_{cg2014}_sgRNA-28	Gibson assembly 1669/1670 (#A *SpeI), circularized	*amp*^{R}, pSM22 derivative with an inserted P_{cg2014}_sgRNA-28 spacer encoding sequence at the SpeI site between the P_{J23119} promoter and the sgRNA hairpin	S: 890	This work
#Y	pSM22-P_{J32119}-P_{cg2014}_sgRNA-29	Gibson assembly 1671/1672 (#A *SpeI), circularized	*amp*^{R}, pSM22 derivative with an inserted P_{cg2014}_sgRNA-29 spacer encoding sequence at the	S: 890	This work

			SpeI site between the P_{J23119} promoter and the sgRNA hairpin		
#Z	pSM22-P_{J32119}-P_{cg2014}_sgRNA-30	Gibson assembly 1673/1674 (#A *SpeI), circularized	*amp*^{R}, pSM22 derivative with an inserted P_{cg2014}_sgRNA-30 spacer encoding sequence at the SpeI site between the P_{J23119} promoter and the sgRNA hairpin	S: 890	This work
#AA	pSM22-P_{J32119}-P_{cg2014}_sgRNA-31	Gibson assembly 1675/1676 (#A *SpeI), circularized	*amp*^{R}, pSM22 derivative with an inserted P_{cg2014}_sgRNA-31 spacer encoding sequence at the SpeI site between the P_{J23119} promoter and the sgRNA hairpin	S: 890	This work
#AB	pJYS3-*dcas12a*		*kan*^{R}, pJYS3 derivative with a *dcas12a* (*cpf1* from *Francisella novicida* carrying mutations D917A and E1006A) sequence under control of the constitutive P_{lacM} promoter		Gift from Lukas Osswald
#AC	pSM22-P_{J23119}-crRNA_SpeI-site	Gibson assembly 1780/1781 (#A	*amp*^{R}, pSM22 derivative containing the P_{J23119} promoter	C: 890/891<br>S: 890	This work

		*SpeI *BamHI), circularized	and the sequence encoding the crRNA hairpin followed by a SpeI site to facilitate spacer insertion (helper plasmid for crRNA amplification)		
#AD	pSM22-P_{J23119}-P_{cg1896}_sgRNA-1	Gibson assembly 852/853 (#A *SpeI), circularized	*amp*^{R}, pSM22 derivative with an inserted P_{cg1896}_sgRNA-1 spacer encoding sequence at the SpeI site between the P_{J23119} promoter and the sgRNA hairpin	S: 890	This work
#AE	pSM22-P_{J23119}-P_{cg1896}_sgRNA-2	Gibson assembly 854/855 (#A *SpeI), circularized	*amp*^{R}, pSM22 derivative with an inserted P_{cg1896}_sgRNA-2 spacer encoding sequence at the SpeI site between the P_{J23119} promoter and the sgRNA hairpin	S: 890	This work
#AF	pSM22-P_{J23119}-P_{cg1929}_sgRNA-1	Gibson assembly 856/857 (#A *SpeI), circularized	*amp*^{R}, pSM22 derivative with an inserted P_{cg1929}_sgRNA-1 spacer encoding sequence at the SpeI site between the P_{J23119} promoter and the sgRNA hairpin	S: 890	This work

#AG	pSM22-P_{J23119}-P_{cg1940}_sgRNA-1	Gibson assembly 858/859 (#A *SpeI), circularized	*amp*^{R}, pSM22 derivative with an inserted P_{cg1940}_sgRNA-1 spacer encoding sequence at the SpeI site between the P_{J23119} promoter and the sgRNA hairpin	S: 890	This work
#AH	pSM22-P_{J23119}-P_{cg1940}_sgRNA-2	Gibson assembly 860/861 (#A *SpeI), circularized	*amp*^{R}, pSM22 derivative with an inserted P_{cg1940}_sgRNA-2 spacer encoding sequence at the SpeI site between the P_{J23119} promoter and the sgRNA hairpin	S: 890	This work
#AJ	pSM22-P_{J23119}-P_{cg1977}_sgRNA-1	Gibson assembly 866/867 (#A *SpeI), circularized	*amp*^{R}, pSM22 derivative with an inserted P_{cg1977}_sgRNA-1 spacer encoding sequence at the SpeI site between the P_{J23119} promoter and the sgRNA hairpin	S: 890	This work
#AK	pSM22-P_{J23119}-P_{cg1999}_sgRNA-1	Gibson assembly 868/869 (#A *SpeI), circularized	*amp*^{R}, pSM22 derivative with an inserted P_{cg1999}_sgRNA-1 spacer encoding sequence at the SpeI site between the P_{J23119} promoter	S: 890	This work

			and the sgRNA hairpin		
#AL	pSM22-P_{J23119}-P_{cg1999}_sgRNA-2	Gibson assembly 870/871 (#A *SpeI), circularized	*amp^R^*, pSM22 derivative with an inserted P_{cg1999}_sgRNA-2 spacer encoding sequence at the SpeI site between the P_{J23119} promoter and the sgRNA hairpin	S: 890	This work
#AM	pSM22-P_{J23119}-P_{cg2004}_sgRNA-1	Gibson assembly 872/873 (#A *SpeI), circularized	*amp^R^*, pSM22 derivative with an inserted P_{cg2004}_sgRNA-1 spacer encoding sequence at the SpeI site between the P_{J23119} promoter and the sgRNA hairpin	S: 890	This work
#AN	pSM22-P_{J23119}-P_{cg2004}_sgRNA-2	Gibson assembly 874/875 (#A *SpeI), circularized	*amp^R^*, pSM22 derivative with an inserted P_{cg2004}_sgRNA-2 spacer encoding sequence at the SpeI site between the P_{J23119} promoter and the sgRNA hairpin	S: 890	This work
#AO	pSM22-P_{J23119}-P_{cg2011}_sgRNA-1	Gibson assembly 876/877 (#A *SpeI), circularized	*amp^R^*, pSM22 derivative with an inserted P_{cg2011}_sgRNA-1 spacer encoding sequence at the	S: 890	This work

			SpeI site between the P_{J23119} promoter and the sgRNA hairpin		
#AP	pSM22-P_{J23119}-P_{cg2014}_sgRNA-1	Gibson assembly 878/879 (#A *SpeI), circularized	*amp^R^*, pSM22 derivative with an inserted P_{cg2014}_sgRNA-1 spacer encoding sequence at the SpeI site between the P_{J23119} promoter and the sgRNA hairpin	S: 890	This work
#AQ	pSM22-P_{J23119}-P_{cg2032}_sgRNA-1	Gibson assembly 880/881 (#A *SpeI), circularized	*amp^R^*, pSM22 derivative with an inserted P_{cg2032}_sgRNA-1 spacer encoding sequence at the SpeI site between the P_{J23119} promoter and the sgRNA hairpin	S: 890	This work
#AR	pSM22-P_{J23119}-P_{cg2032}_sgRNA-2	Gibson assembly 882/883 (#A *SpeI), circularized	*amp^R^*, pSM22 derivative with an inserted P_{cg2032}_sgRNA-2 spacer encoding sequence at the SpeI site between the P_{J23119} promoter and the sgRNA hairpin	S: 890	This work
#AS	pSM22-P_{J23119}-P_{cg2065}_sgRNA-1	Gibson assembly 884/885 (#A	*amp^R^*, pSM22 derivative with an inserted P_{cg2065}_sgRNA-1	S: 890	This work

		*SpeI), circularized	spacer encoding sequence at the SpeI site between the P_{J23119} promoter and the sgRNA hairpin		
#AT	#125687 (Addgene)<br>pCRISPR-dCas9		*apr^R^, E. coli* vector containing *Streptomyces* codon optimized spdCas9, sgRNA cassette		(15)
#AU	pMC386		*thio^R^, E. coli* vector containing theophylline riboswitch controlled *tipA*		Gift from Meghan Pepler
#AV	pSET152		*apr^R^, E. coli-Streptomyces* shuttle vector containing a phiC31 *attP-int* cassette		(16)
#1	pEC-XC99E-*lacI*-P_{tac}-*dcas9*_no-sgRNA	Gibson assembly: R87/R88 (#b) into #C *SpeI *XmaI	*cm^R^*, pEC-XC99E-*dcas9*-sgRNA_hairpin-T_{Sp} (#C) derivative with an inserted *lacI*-P_{tac} sequence upstream of the *dcas9* gene	C: R95/R96<br>S: R171, R169	This work
#2	pEC-XC99E-*lacI*-P_{tac}-*dcas9*--$P_{tac\Delta}$-sgRNA-CS3	Gibson assembly: annealed R176/R177 dsDNA into #D *Bsp1407I *SpeI	*cm^R^*, pEC-XC99E-*lacI*-P_{tac}-*dcas9*--$P_{tac\Delta}$-SpeI_site-sgRNA_hairpin-T_{Sp} (#D) derivative with an inserted P_{cg1974}_sgRNA-CS3 spacer encoding	S: R171	This work

			sequence at the SpeI site		
#3	pJC1-P_{cg1974}-*venus*		*kan*^{R}, pJC1-based plasmid carrying the CgpS bound area of the promoter of cg1974 (444 bp) and the first 30 bp of the coding sequence fused to the reporter gene *venus* via a linker containing a stop codon and an artificial RBS		(5) (pJC1-P_{lys}-*venus*)
#4	pEC-XC99E_empty	Gibson assembly annealed R65/R66 dsDNA into #a *NdeI *SalI	*cm*^{R}, pEC-XC99E (#a) derivative lacking the *lacI*^{q} gene and the P_{trc} promoter	C: R71/R72<br>S: R71	This work
#5	pEC-XC99E-*lacI*-P_{tac}-*dcas9*--$P_{tac\Delta}$-sgRNA-CS1	Gibson assembly annealed R172/R173 dsDNA into #D *Bsp1407I *SpeI	*cm*^{R}, pEC-XC99E-*lacI*-P_{tac}-*dcas9*--$P_{tac\Delta}$-SpeI_site-sgRNA_hairpin-T_{Sp} (#D) derivative with an inserted P_{cg1974}_sgRNA-CS1 spacer encoding sequence at the SpeI site	S: R171	This work
#6	pJC1-P_{cg1974}-*venus*_T_P_{J23119}-sgRNA-CS3	Gibson assembly 1341/1342 (#E) into #3 *SalI	*kan*^{R}, pJC1-P_{cg1974}-*venus* (#3) derivative with an inserted P_{cg1974}_sgRNA-CS3 sequence under	C: 766/R13<br>S: R13	This work

			control of the P_{J23119} promoter and followed by the *rrnB* terminator behind the *venus*-*rrnB* (T) sequence		
#7	pJC1-$P_{cg1974\Delta PAM}$-*venus*	Gibson assembly 117/B024 (#3) and B026/116 (#3) into #74 *BamHI *SpeI	*kan^R*, pJC1-P_{cg1974}-*venus* (#3) derivative with a deleted sgRNA-CS3 specific PAM sequence in P_{cg1974} (change of AGG to AAC 39-37 bp upstream of gene start)	C: R12/R13<br>S: R12, R13	This work
#8	pJC1-P_{cg2014}-*venus*		*kan^R*, pJC1-based plasmid carrying the CgpS bound area of the promoter of cg2014 (545 bp) and the first 30 bp of the coding sequence fused to the reporter gene *venus* via a linker containing a stop codon and an artificial RBS		(5)
#9	pJC1-P_{cg1974}-*venus*_T_P_{J23119}-sgRNA-25	Gibson assembly 1341/1342 (#F) into #3 *SalI	*kan^R*, pJC1-P_{cg1974}-*venus* (#3) derivative with an inserted P_{cg1974}_sgRNA-25 sequence under control of the P_{J23119} promoter and followed by the	C: 554/R13<br>S: 554, R13	This work

			rrnB terminator behind the *venus*-*rrnB* (T) sequence		
#10	pJC1-P_{cg1974}-*venus*_T_P_{J23119}-sgRNA-26	Gibson assembly 1341/1342 (#G) into #3 *SalI	*kan*^{R}, pJC1-P_{cg1974}-*venus* (#3) derivative with an inserted P_{cg1974}_sgRNA-26 sequence under control of the P_{J23119} promoter and followed by the *rrnB* terminator behind the *venus*-*rrnB* (T) sequence	C: 554/R13<br>S: 554, R13	This work
#11	pJC1-P_{cg1974}-*venus*_T_P_{J23119}-sgRNA-28	Gibson assembly 1341/1342 (#H) into #3 *SalI	*kan*^{R}, pJC1-P_{cg1974}-*venus* (#3) derivative with an inserted P_{cg1974}_sgRNA-28 sequence under control of the P_{J23119} promoter and followed by the *rrnB* terminator behind the *venus*-*rrnB* (T) sequence	C: 554/R13<br>S: 554, R13	This work
#12	pJC1-P_{cg1974}-*venus*_T_P_{J23119}-sgRNA-29	Gibson assembly 1341/1342 (#I) into #3 *SalI	*kan*^{R}, pJC1-P_{cg1974}-*venus* (#3) derivative with an inserted P_{cg1974}_sgRNA-29 sequence under control of the P_{J23119} promoter and followed by the *rrnB* terminator	C: 554/R13<br>S: 554, R13	This work

			behind the *venus*-*rrnB* (T) sequence		
#13	pJC1-P_{cg1974}-*venus*_T_P_{J23119}-sgRNA-CS1	Gibson assembly 1341/1342 (#J) into #3 *SalI	*kan*^{R}, pJC1-P_{cg1974}-*venus* (#3) derivative with an inserted P_{cg1974}_sgRNA-CS1 sequence under control of the P_{J23119} promoter and followed by the *rrnB* terminator behind the *venus*-*rrnB* (T) sequence	C: 554/R13<br>S: 554, R13	This work
#15	pJC1-P_{cg1974}-*venus*_T_P_{J23119}-sgRNA-33	Gibson assembly 1341/1342 (#K) into #3 *SalI	*kan*^{R}, pJC1-P_{cg1974}-*venus* (#3) derivative with an inserted P_{cg1974}_sgRNA-33 sequence under control of the P_{J23119} promoter and followed by the *rrnB* terminator behind the *venus*-*rrnB* (T) sequence	C: 554/R13<br>S: 554, R13	This work
#16	pJC1-P_{cg1974}-*venus*_T_P_{J23119}-sgRNA-34	Gibson assembly 1341/1342 (#L) into #3 *SalI	*kan*^{R}, pJC1-P_{cg1974}-*venus* (#3) derivative with an inserted P_{cg1974}_sgRNA-34 sequence under control of the P_{J23119} promoter and followed by the *rrnB* terminator behind the *venus*-*rrnB* (T) sequence	C: 554/R13<br>S: 554, R13	This work

#17	pJC1-P_{cg1974}-*venus*_T_P_{J23119}-sgRNA-35	Gibson assembly 1341/1342 (#M) into #3 *SalI	*kan*^{R}, pJC1-P_{cg1974}-*venus* (#3) derivative with an inserted P_{cg1974}_sgRNA-35 sequence under control of the P_{J23119} promoter and followed by the *rrnB* terminator behind the *venus*-*rrnB* (T) sequence	C: 554/R13<br>S: 554, R13	This work
#18	pJC1-P_{cg1974}-*venus*_T_P_{J23119}-sgRNA-36	Gibson assembly 1341/1342 (#N) into #3 *SalI	*kan*^{R}, pJC1-P_{cg1974}-*venus* (#3) derivative with an inserted P_{cg1974}_sgRNA-36 sequence under control of the P_{J23119} promoter and followed by the *rrnB* terminator behind the *venus*-*rrnB* (T) sequence	C: 554/R13<br>S: 554, R13	This work
#19	pJC1-P_{cg1974}-*venus*_T_P_{J23119}-sgRNA-37	Gibson assembly 1341/1342 (#O) into #3 *SalI	*kan*^{R}, pJC1-P_{cg1974}-*venus* (#3) derivative with an inserted P_{cg1974}_sgRNA-37 sequence under control of the P_{J23119} promoter and followed by the *rrnB* terminator behind the *venus*-*rrnB* (T) sequence	C: 554/R13<br>S: 554, R13	This work

#20	pJC1-P_{cg1974}-*venus*_T_P_{J23119}-sgRNA-38	Gibson assembly 1341/1342 (#P) into #3 *SalI	*kan*^{R}, pJC1-P_{cg1974}-*venus* (#3) derivative with an inserted P_{cg1974}_sgRNA-38 sequence under control of the P_{J23119} promoter and followed by the *rrnB* terminator behind the *venus*-*rrnB* (T) sequence	C: 554/R13<br>S: 554, R13	This work
#21	pJC1-P_{cg1974}-*venus*_T_P_{J23119}-sgRNA-39	Gibson assembly 1341/1342 (#Q) into #3 *SalI	*kan*^{R}, pJC1-P_{cg1974}-*venus* (#3) derivative with an inserted P_{cg1974}_sgRNA-39 sequence under control of the P_{J23119} promoter and followed by the *rrnB* terminator behind the *venus*-*rrnB* (T) sequence	C: 554/R13<br>S: 554, R13	This work
#22	pJC1-P_{cg1974}-*venus*_T_P_{J23119}-sgRNA-40	Gibson assembly 1341/1342 (#R) into #3 *SalI	*kan*^{R}, pJC1-P_{cg1974}-*venus* (#3) derivative with an inserted P_{cg1974}_sgRNA-40 sequence under control of the P_{J23119} promoter and followed by the *rrnB* terminator behind the *venus*-*rrnB* (T) sequence	C: 554/R13<br>S: 554, R13	This work

#23	pJC1-P_{cg2014}-*venus*_T_P_{J23119}-sgRNA-22	Gibson assembly 1341/1342 (#S) into #8 *SalI	*kan*^{R}, pJC1-P_{cg2014}-*venus* (#8) derivative with an inserted P_{cg2014}_sgRNA-22 sequence under control of the P_{J23119} promoter and followed by the *rrnB* terminator behind the *venus*-*rrnB* (T) sequence	C: 554/R13<br>S: 554, R13	This work
#24	pJC1-P_{cg2014}-*venus*_T_P_{J23119}-sgRNA-24	Gibson assembly 1341/1342 (#T) into #8 *SalI	*kan*^{R}, pJC1-P_{cg2014}-*venus* (#8) derivative with an inserted P_{cg2014}_sgRNA-24 sequence under control of the P_{J23119} promoter and followed by the *rrnB* terminator behind the *venus*-*rrnB* (T) sequence	C: 554/R13<br>S: 554, R13	This work
#25	pJC1-P_{cg2014}-*venus*_T_P_{J23119}-sgRNA-25	Gibson assembly 1341/1342 (#U) into #8 *SalI	*kan*^{R}, pJC1-P_{cg2014}-*venus* (#8) derivative with an inserted P_{cg2014}_sgRNA-25 sequence under control of the P_{J23119} promoter and followed by the *rrnB* terminator behind the *venus*-*rrnB* (T) sequence	C: 554/R13<br>S: 554, R13	This work

#26	pJC1-P_{cg2014}-*venus*_T_P_{J23119}-sgRNA-26	Gibson assembly 1341/1342 (#V) into #8 *SalI	*kan*^{R}, pJC1-P_{cg2014}-*venus* (#8) derivative with an inserted P_{cg2014}_sgRNA-26 sequence under control of the P_{J23119} promoter and followed by the *rrnB* terminator behind the *venus*-*rrnB* (T) sequence	C: 554/R13<br>S: 554, R13	This work
#27	pJC1-P_{cg2014}-*venus*_T_P_{J23119}-sgRNA-27	Gibson assembly 1341/1342 (#W) into #8 *SalI	*kan*^{R}, pJC1-P_{cg2014}-*venus* (#8) derivative with an inserted P_{cg2014}_sgRNA-27 sequence under control of the P_{J23119} promoter and followed by the *rrnB* terminator behind the *venus*-*rrnB* (T) sequence	C: 554/R13<br>S: 554, R13	This work
#28	pJC1-P_{cg2014}-*venus*_T_P_{J23119}-sgRNA-28	Gibson assembly 1341/1342 (#X) into #8 *SalI	*kan*^{R}, pJC1-P_{cg2014}-*venus* (#8) derivative with an inserted P_{cg2014}_sgRNA-28 sequence under control of the P_{J23119} promoter and followed by the *rrnB* terminator behind the *venus*-*rrnB* (T) sequence	C: 554/R13<br>S: 554, R13	This work

#29	pJC1-P_{cg2014}-*venus*_T_P_{J23119}-sgRNA-29	Gibson assembly 1341/1342 (#Y) into #8 *SalI	*kan^R^*, pJC1-P_{cg2014}-*venus* (#8) derivative with an inserted P_{cg2014}_sgRNA-29 sequence under control of the P_{J23119} promoter and followed by the *rrnB* terminator behind the *venus*-*rrnB* (T) sequence	C: 554/R13<br>S: 554, R13	This work
#30	pJC1-P_{cg2014}-*venus*_T_P_{J23119}-sgRNA-30	Gibson assembly 1341/1342 (#Z) into #8 *SalI	*kan^R^*, pJC1-P_{cg2014}-*venus* (#8) derivative with an inserted P_{cg2014}_sgRNA-30 sequence under control of the P_{J23119} promoter and followed by the *rrnB* terminator behind the *venus*-*rrnB* (T) sequence	C: 554/R13<br>S: 554, R13	This work
#31	pJC1-P_{cg2014}-*venus*_T_P_{J23119}-sgRNA-31	Gibson assembly 1341/1342 (#AA) into #8 *SalI	*kan^R^*, pJC1-P_{cg2014}-*venus* (#8) derivative with an inserted P_{cg2014}_sgRNA-31 sequence under control of the P_{J23119} promoter and followed by the *rrnB* terminator behind the *venus*-*rrnB* (T) sequence	C: 554/R13<br>S: 554, R13	This work

#32	pJC1-P_{cg1974}-*venus*_T_P_{lacM}-*dcas12a*	Gibson assembly 1778/1779 (#AB) into #3 *SalI	*kan*^{R}, pJC1-P_{cg1974}-*venus* (#3) derivative with an inserted *dcas12a* (*cpf1* from *Francisella novicida* carrying mutations D917A and E1006A) sequence under control of the constitutive P_{lacM} promoter and followed by the *rrnB* terminator behind the *venus*-*rrnB* (T) sequence	C: 1788/1792<br>S: 1787, 1788, 1789, 1790, 1791, 1792	This work
#33	pEC-XC99E_P_{J23119}-P_{cg1974}_crRNA-7	Gibson assembly 1782/1800 (#AC *SpeI) into 1873/1874 (#4 *SdaI)	*cm*^{R}, pEC-XC99E_empty (#4) derivative with an inserted P_{cg1974}_crRNA-7 sequence under control of the P_{J23119} promoter in front of the *rrnB* terminator	C: 1782/1442<br>S: 1441	This work
#34	pEC-XC99E_P_{J23119}-P_{cg1974}_crRNA-1	Gibson assembly 1782/1783 (#AC *SpeI) into 1873/1874 (#4 *SdaI)	*cm*^{R}, pEC-XC99E_empty (#4) derivative with an inserted P_{cg1974}_crRNA-1 sequence under control of the P_{J23119} promoter in front of the *rrnB* terminator	C: 1782/1442<br>S: 1441	This work
#35	pEC-XC99E_P_{J23119}-P_{cg1974}_crRNA-3	Gibson assembly 1782/1796 (#AC *SpeI) into	*cm*^{R}, pEC-XC99E_empty (#4) derivative with an inserted P_{cg1974}_crRNA-3	C: 1782/1442<br>S: 1441	This work

		1873/1874 (#4 *SdaI)	sequence under control of the P_{J23119} promoter in front of the *rrnB* terminator		
#36	pEC-XC99E_P_{J23119}-P_{cg1974}_crRNA-4	Gibson assembly 1782/1797 (#AC *SpeI) into 1873/1874 (#4 *SdaI)	*cm*^{R}, pEC-XC99E_empty (#4) derivative with an inserted P_{cg1974}_crRNA-4 sequence under control of the P_{J23119} promoter in front of the *rrnB* terminator	C: 1782/1442<br>S: 1441	This work
#37	pEC-XC99E_P_{J23119}-P_{cg1974}_crRNA-5	Gibson assembly 1782/1798 (#AC *SpeI) into 1873/1874 (#4 *SdaI)	*cm*^{R}, pEC-XC99E_empty (#4) derivative with an inserted P_{cg1974}_crRNA-5 sequence under control of the P_{J23119} promoter in front of the *rrnB* terminator	C: 1782/1442<br>S: 1441	This work
#38	pEC-XC99E_P_{J23119}-P_{cg1974}_crRNA-6	Gibson assembly 1782/1799 (#AC *SpeI) into 1873/1874 (#4 *SdaI)	*cm*^{R}, pEC-XC99E_empty (#4) derivative with an inserted P_{cg1974}_crRNA-6 sequence under control of the P_{J23119} promoter in front of the *rrnB* terminator	C: 1782/1442<br>S: 1441	This work
#39	pEC-XC99E_P_{J23119}-P_{cg1974}_crRNA-8	Gibson assembly 1782/1801 (#AC *SpeI) into 1873/1874 (#4 *SdaI)	*cm*^{R}, pEC-XC99E_empty (#4) derivative with an inserted P_{cg1974}_crRNA-8 sequence under control of the P_{J23119}	C: 1782/1442<br>S: 1441	This work

			promoter in front of the *rrnB* terminator		
#40	pEC-XC99E_P_{J23119}-P_{cg1974}_crRNA-9	Gibson assembly 1782/1802 (#AC *SpeI) into 1873/1874 (#4 *SdaI)	*cm*^{R}, pEC-XC99E_empty (#4) derivative with an inserted P_{cg1974}_crRNA-9 sequence under control of the P_{J23119} promoter in front of the *rrnB* terminator	C: 1782/1442<br>S: 1441	This work
#41	pEC-XC99E_P_{J23119}-P_{cg1974}_crRNA-10	Gibson assembly 1782/1803 (#AC *SpeI) into 1873/1874 (#4 *SdaI)	*cm*^{R}, pEC-XC99E_empty (#4) derivative with an inserted P_{cg1974}_crRNA-10 sequence under control of the P_{J23119} promoter in front of the *rrnB* terminator	C: 1782/1442<br>S: 1441	This work
#42	pJC1-P_{cg2014}-*venus*_T_P_{lacM}-*dcas12a*	Gibson assembly 1778/1779 (#AB) into #8 *SalI	*kan*^{R}, pJC1-P_{cg2014}-*venus* (#8) derivative with an inserted *dcas12a* (*cpf1* from *Francisella novicida* carrying mutations D917A and E1006A) sequence under control of the constitutive P_{lacM} promoter and followed by the *rrnB* terminator behind the *venus*-*rrnB* (T) sequence	C: 1788/1792<br>S: 1787, 1788, 1789, 1790, 1791, 1792	This work

#43	pEC-XC99E_P_{J23119}-P_{cg2014}_crRNA-1	Gibson assembly 1782/1875 (#AC *SpeI) into 1873/1874 (#4 *SdaI)	*cm*^{R}, pEC-XC99E_empty (#4) derivative with an inserted P_{cg2014}_crRNA-1 sequence under control of the P_{J23119} promoter in front of the *rrnB* terminator	C: 1782/1442<br>S: 1441	This work
#44	pEC-XC99E_P_{J23119}-P_{cg2014}_crRNA-2	Gibson assembly 1782/1876 (#AC *SpeI) into 1873/1874 (#4 *SdaI)	*cm*^{R}, pEC-XC99E_empty (#4) derivative with an inserted P_{cg2014}_crRNA-2 sequence under control of the P_{J23119} promoter in front of the *rrnB* terminator	C: 1782/1442<br>S: 1441	This work
#45	pEC-XC99E_P_{J23119}-P_{cg2014}_crRNA-3	Gibson assembly 1782/1877 (#AC *SpeI) into 1873/1874 (#4 *SdaI)	*cm*^{R}, pEC-XC99E_empty (#4) derivative with an inserted P_{cg2014}_crRNA-3 sequence under control of the P_{J23119} promoter in front of the *rrnB* terminator	C: 1782/1442<br>S: 1441	This work
#46	pEC-XC99E_P_{J23119}-P_{cg2014}_crRNA-4	Gibson assembly 1782/1878 (#AC *SpeI) into 1873/1874 (#4 *SdaI)	*cm*^{R}, pEC-XC99E_empty (#4) derivative with an inserted P_{cg2014}_crRNA-4 sequence under control of the P_{J23119} promoter in front of the *rrnB* terminator	C: 1782/1442<br>S: 1441	This work

#47	pEC-XC99E_P_{J23119}-P_{cg2014}_crRNA-6	Gibson assembly 1782/1880 (#AC *SpeI) into 1873/1874 (#4 *SdaI)	*cm^R*, pEC-XC99E_empty (#4) derivative with an inserted P_{cg2014}_crRNA-6 sequence under control of the P_{J23119} promoter in front of the *rrnB* terminator	C: 1782/1442<br>S: 1441	This work
#48	pEC-XC99E_P_{J23119}-P_{cg2014}_crRNA-7	Gibson assembly 1782/1881 (#AC *SpeI) into 1873/1874 (#4 *SdaI)	*cm^R*, pEC-XC99E_empty (#4) derivative with an inserted P_{cg2014}_crRNA-7 sequence under control of the P_{J23119} promoter in front of the *rrnB* terminator	C: 1782/1442<br>S: 1441	This work
#49	pJC1-P_{cg1896}-*venus*	Gibson assembly 789/722 (genomic *C. glutamicum* DNA) and 115/116 (#74) into #74 *BamHI *SpeI	*kan^R*, pJC1-*venus* (#74) derivative carrying the CgpS bound area of the promoter of cg1896 (185 bp) and the first 30 bp of the coding sequence fused to the reporter gene *venus* via a linker containing a stop codon and an artificial RBS	C: R12/R13<br>S: R12, R13	This work
#50	pJC1-P_{cg1929}-*venus*	Gibson assembly 792/723 (genomic *C. glutamicum* DNA) and 115/116 (#74)	*kan^R*, pJC1-*venus* (#74) derivative carrying the CgpS bound area of the promoter of cg1929 (441 bp) and the first 30 bp of the	C: R12/R13<br>S: R12, R13	This work

		into #74 *BamHI *SpeI	coding sequence fused to the reporter gene *venus* via a linker containing a stop codon and an artificial RBS	
#51	pJC1-P_{cg1940}-*venus*		*kan*^{R}, pJC1-based plasmid carrying the CgpS bound area of the promoter of cg1940 (563 bp) and the first 30 bp of the coding sequence fused to the reporter gene *venus* via a linker containing a stop codon and an artificial RBS	(5)
#52	pJC1-P_{cg1977}-*venus*		*kan*^{R}, pJC1-based plasmid carrying the CgpS bound area of the promoter of cg1977 (653 bp) and the first 30 bp of the coding sequence fused to the reporter gene *venus* via a linker containing a stop codon and an artificial RBS	(5)
#53	pJC1-P_{cg1999}-*venus*		*kan*^{R}, pJC1-based plasmid carrying the CgpS bound area of the	(5)

			promoter of cg1999 (448 bp) and the first 30 bp of the coding sequence fused to the reporter gene *venus* via a linker containing a stop codon and an artificial RBS		
#54	pJC1-P_{cg2004}-*venus*	Gibson assembly 790/724 (genomic *C. glutamicum* DNA) and 115/116 (#74) into #74 *BamHI *SpeI	*kan*^{R}, pJC1-*venus* (#74) derivative carrying the CgpS bound area of the promoter of cg2004 (473 bp) and the first 30 bp of the coding sequence fused to the reporter gene *venus* via a linker containing a stop codon and an artificial RBS	C: R12/R13<br>S: R12, R13	This work
#55	pJC1-P_{cg2011}-*venus*	Gibson assembly 791/725 (genomic *C. glutamicum* DNA) and 115/116 (#74) into #74 *BamHI *SpeI	*kan*^{R}, pJC1-*venus* (#74) derivative carrying the CgpS bound area of the promoter of cg2011 (363 bp) and the first 30 bp of the coding sequence fused to the reporter gene *venus* via a linker containing a stop codon and an artificial RBS	C: R12/R13<br>S: R12, R13	This work

#56	pJC1-P_{cg2032}-*venus*		*kan*^{R}, pJC1-based plasmid carrying the CgpS bound area of the promoter of cg2032 (490 bp) and the first 30 bp of the coding sequence fused to the reporter gene *venus* via a linker containing a stop codon and an artificial RBS		(5)
#57	pJC1-P_{cg2065}-*venus*	Gibson assembly 793/726 (genomic *C. glutamicum* DNA) and 115/116 (#74) into #74 *BamHI *SpeI	*kan*^{R}, pJC1-*venus* (#74) derivative carrying the CgpS bound area of the promoter of cg2065 (480 bp) and the first 30 bp of the coding sequence fused to the reporter gene *venus* via a linker containing a stop codon and an artificial RBS	C: R12/R13<br>S: R12, R13	This work
#58	pJC1-P_{cg1896}-*venus*_T_P_{J23119}-sgRNA-1	Gibson assembly 1341/1342 (#AD) into #49 *SalI	*kan*^{R}, pJC1-P_{cg1896}-*venus* (#49) derivative with an inserted P_{cg1896}_sgRNA-1 sequence under control of the P_{J23119} promoter and followed by the *rrnB* terminator	C: 554/R13<br>S: 554, R13	This work

			behind the *venus*-*rrnB* (T) sequence		
#59	pJC1-P_{cg1896}-*venus*_T_P_{J23119}-sgRNA-2	Gibson assembly 1341/1342 (#AE) into #49 *SalI	*kan*^{R}, pJC1-P_{cg1896}-*venus* (#49) derivative with an inserted P_{cg1896}_sgRNA-2 sequence under control of the P_{J23119} promoter and followed by the *rrnB* terminator behind the *venus*-*rrnB* (T) sequence	C: 554/R13<br>S: 554, R13	This work
#60	pJC1-P_{cg1929}-*venus*_T_P_{J23119}-sgRNA-1	Gibson assembly 1341/1342 (#AF) into #50 *SalI	*kan*^{R}, pJC1-P_{cg1929}-*venus* (#50) derivative with an inserted P_{cg1929}_sgRNA-1 sequence under control of the P_{J23119} promoter and followed by the *rrnB* terminator behind the *venus*-*rrnB* (T) sequence	C: 554/R13<br>S: 554, R13	This work
#61	pJC1-P_{cg1940}-*venus*_T_P_{J23119}-sgRNA-1	Gibson assembly 1341/1342 (#AG) into #51 *SalI	*kan*^{R}, pJC1-P_{cg1940}-*venus* (#51) derivative with an inserted P_{cg1940}_sgRNA-1 sequence under control of the P_{J23119} promoter and followed by the *rrnB* terminator behind the *venus*-*rrnB* (T) sequence	C: 554/R13<br>S: 554, R13	This work

#62	pJC1-P_{cg1940}-*venus*_T_P_{J23119}-sgRNA-2	Gibson assembly 1341/1342 (#AH) into #51 *SalI	*kan^R*, pJC1-P_{cg1940}-*venus* (#51) derivative with an inserted P_{cg1940}_sgRNA-2 sequence under control of the P_{J23119} promoter and followed by the *rrnB* terminator behind the *venus*-*rrnB* (T) sequence	C: 554/R13<br>S: 554, R13	This work
#64	pJC1-P_{cg1977}-*venus*_T_P_{J23119}-sgRNA-1	Gibson assembly 1341/1342 (#AJ) into #52 *SalI	*kan^R*, pJC1-P_{cg1977}-*venus* (#52) derivative with an inserted P_{cg1977}_sgRNA-1 sequence under control of the P_{J23119} promoter and followed by the *rrnB* terminator behind the *venus*-*rrnB* (T) sequence	C: 554/R13<br>S: 554, R13	This work
#65	pJC1-P_{cg1999}-*venus*_T_P_{J23119}-sgRNA-1	Gibson assembly 1341/1342 (#AK) into #53 *SalI	*kan^R*, pJC1-P_{cg1999}-*venus* (#53) derivative with an inserted P_{cg1999}_sgRNA-1 sequence under control of the P_{J23119} promoter and followed by the *rrnB* terminator behind the *venus*-*rrnB* (T) sequence	C: 554/R13<br>S: 554, R13	This work

#66	pJC1-P_{cg1999}-*venus*_T_P_{J23119}-sgRNA-2	Gibson assembly 1341/1342 (#AL) into #53 *SalI	*kan*^{R}, pJC1-P_{cg1999}-*venus* (#53) derivative with an inserted P_{cg1999}_sgRNA-2 sequence under control of the P_{J23119} promoter and followed by the *rrnB* terminator behind the *venus*-*rrnB* (T) sequence	C: 554/R13<br>S: 554, R13	This work
#67	pJC1-P_{cg2004}-*venus*_T_P_{J23119}-sgRNA-1	Gibson assembly 1341/1342 (#AM) into #54 *SalI	*kan*^{R}, pJC1-P_{cg2004}-*venus* (#54) derivative with an inserted P_{cg2004}_sgRNA-1 sequence under control of the P_{J23119} promoter and followed by the *rrnB* terminator behind the *venus*-*rrnB* (T) sequence	C: 554/R13<br>S: 554, R13	This work
#68	pJC1-P_{cg2004}-*venus*_T_P_{J23119}-sgRNA-2	Gibson assembly 1341/1342 (#AN) into #54 *SalI	*kan*^{R}, pJC1-P_{cg2004}-*venus* (#54) derivative with an inserted P_{cg2004}_sgRNA-2 sequence under control of the P_{J23119} promoter and followed by the *rrnB* terminator behind the *venus*-*rrnB* (T) sequence	C: 554/R13<br>S: 554, R13	This work

#69	pJC1-P_{cg2011}-*venus*_T_P_{J23119}-sgRNA-1	Gibson assembly 1341/1342 (#AO) into #55 *SalI	*kan*^{R}, pJC1-P_{cg2011}-*venus* (#55) derivative with an inserted P_{cg2011}_sgRNA-1 sequence under control of the P_{J23119} promoter and followed by the *rrnB* terminator behind the *venus-rrnB* (T) sequence	C: 554/R13<br>S: 554, R13	This work
#70	pJC1-P_{cg2014}-*venus*_T_P_{J23119}-sgRNA-1	Gibson assembly 1341/1342 (#AP) into #8 *SalI	*kan*^{R}, pJC1-P_{cg2014}-*venus* (#8) derivative with an inserted P_{cg2014}_sgRNA-1 sequence under control of the P_{J23119} promoter and followed by the *rrnB* terminator behind the *venus-rrnB* (T) sequence	C: 554/R13<br>S: 554, R13	This work
#71	pJC1-P_{cg2032}-*venus*_T_P_{J23119}-sgRNA-1	Gibson assembly 1341/1342 (#AQ) into #56 *SalI	*kan*^{R}, pJC1-P_{cg2032}-*venus* (#56) derivative with an inserted P_{cg2032}_sgRNA-1 sequence under control of the P_{J23119} promoter and followed by the *rrnB* terminator behind the *venus-rrnB* (T) sequence	C: 554/R13<br>S: 554, R13	This work

#72	pJC1-P_{cg2032}-*venus*_T_P_{J23119}-sgRNA-2	Gibson assembly 1341/1342 (#AR) into #56 *SalI	*kan*^{R}, pJC1-P_{cg2032}-*venus* (#56) derivative with an inserted P_{cg2032}_sgRNA-2 sequence under control of the P_{J23119} promoter and followed by the *rrnB* terminator behind the *venus*-*rrnB* (T) sequence	C: 554/R13<br>S: 554, R13	This work
#73	pJC1-P_{cg2065}-*venus*_T_P_{J23119}-sgRNA-1	Gibson assembly 1341/1342 (#AS) into #57 *SalI	*kan*^{R}, pJC1-P_{cg2065}-*venus* (#57) derivative with an inserted P_{cg2065}_sgRNA-1 sequence under control of the P_{J23119} promoter and followed by the *rrnB* terminator behind the *venus*-*rrnB* (T) sequence	C: 554/R13<br>S: 554, R13	This work
#74	pJC1-*venus*		*kan*^{R}, *E. coli*/*C. glutamicum* shuttle vector (pCG1 ori_{Cg}, pACYC177 p15A ori_{Ec}, promoter-less *venus* gene followed by terminator sequence from *Bacillus subtilis*)		(17)<br>(pJC1-*venus*-term)
#75	pJC1-P_{LAC}_tandem-P_{cg1974}-*venus*	1494/116 (#3 *BamHI)	*kan*^{R}, pJC1-*venus* (#74) derivative with an inserted P_{LAC} promoter	C: 766/114<br>S: 1491, R12, 555, R13	This work

		Into #74 *BamHI *SpeI	sequence in tandem orientation to the P_{cg1974} promoter sequence that was fused to the reporter gene *venus* via a linker containing a stop codon and an artificial RBS		
#76	pJC1-P_{J23150}_tandem-P_{cg1974}-*venus*	1493/116 (#3 *BamHI)<br>Into #74 *BamHI *SpeI	*kan*^{R}, pJC1-*venus* (#74) derivative with an inserted P_{J23150} promoter sequence in tandem orientation to the P_{cg1974} promoter sequence that was fused to the reporter gene *venus* via a linker containing a stop codon and an artificial RBS	C: 766/114<br>S: 1491, R12, 555, R13	This work
#77	pJC1-P_{J23119}_tandem-P_{cg1974}-*venus*	1492/116 (#3 *BamHI)<br>Into #74 *BamHI *SpeI	*kan*^{R}, pJC1-*venus* (#74) derivative with an inserted P_{J23119} promoter sequence in tandem orientation to the P_{cg1974} promoter sequence that was fused to the reporter gene *venus* via a linker containing a stop codon and an artificial RBS	C: 766/114<br>S: 1491, R12, 555, R13	This work

#78	pJC1-P_{LAC}_divergent-P_{cg1974}-*venus*	1497/851 (#A *SpeI)<br>Into #3 *BamHI	*kan^R*, pJC1-*venus* (#74) derivative with an inserted P_{LAC} promoter sequence in divergent orientation to the P_{cg1974} promoter sequence that was fused to the reporter gene *venus* via a linker containing a stop codon and an artificial RBS	C: R12/492<br>S: 1491, R12, 492	This work
#79	pJC1-P_{J23150}_divergent-P_{cg1974}-*venus*	1496/851 (#A *SpeI)<br>Into #3 *BamHI	*kan^R*, pJC1-*venus* (#74) derivative with an inserted P_{J23150} promoter sequence in divergent orientation to the P_{cg1974} promoter sequence that was fused to the reporter gene *venus* via a linker containing a stop codon and an artificial RBS	C: R12/492<br>S: 1491, R12, 492	This work
#80	pJC1-P_{J23119}_divergent-P_{cg1974}-*venus*	848/851 (#A *SpeI)<br>Into #3 *BamHI	*kan^R*, pJC1-*venus* (#74) derivative with an inserted P_{J23119} promoter sequence in divergent orientation to the P_{cg1974} promoter sequence that was fused to the	C: R12/492<br>S: 1491, R12, 492	This work

			reporter gene *venus* via a linker containing a stop codon and an artificial RBS		
#81	pJC1-P_{J23119}_forward-*venus*	1424/1422 (#74) and 1423/1342 (#A) into #74 *SalI	*kan*^{R}, pJC1-*venus* (#74) derivative with an inserted P_{J23119} promoter sequence fused in forward orientation to the reporter gene *venus* via a linker containing an artificial RBS	C: R12/R13<br>S: R12, 492, R13	This work
#82	pJC1-P_{J23119}_reverse-*venus*	1527/851 (#A) and 115/116 (#74) into #74 *BamHI *SpeI	*kan*^{R}, pJC1-*venus* (#74) derivative with an inserted P_{J23119} promoter sequence combined with the *rrnB* terminator in reverse orientation to the reporter gene *venus* via a linker containing an artificial RBS	C: R12/R13<br>S: 1491, R12, 555, R13	This work
#83	pMC345	Gibson assembly: three successive fragment additions into #AT: (i) dCas9-M13F/dCas9-TipAR (#AU) *StuI *BglII, (ii) CRISPR-pSET152-1/CRISPR-	*apr*^{R}, *thio*^{R} pCRISPR-dCas9 (#AT) derivative with theophylline riboswitch-controlled *tipA,* integration apparatus (phage C31 *attP-int* locus), and a new, unique	S: whole plasmid sequencing by plasmidsaurus	This work

		pSET152-2 (#AV) *ApaLI *NotI, (iii) FixNco-F3, FixNco-R/FixNco-R2 (#AT) *AseI *SnaBI	*Avr*II site for guide sequence cloning		
#84	pMC346	Gibson assembly: guide sequence $P_{vnz04460}$_sgRNA into #83 *AvrII	*apr^R*, *$thio^R$* pMC345 (#83) derivative with $P_{vnz04460}$_sgRNA	S: universal M13 Reverse	This work
#85	pMC347	Gibson assembly: guide sequence *vnz04465*_sgRNA into #83 *AvrII	*apr^R*, *$thio^R$* pMC345 (#83) derivative with *vnz04465*_sgRNA	S: universal M13 Reverse	This work

Table S3A. Oligonucleotides used for plasmid constructions (see Table S2).

**Oligonucleotide**	**Sequence (5´-3´)**
B024	AGTTTAATTTGTAGTATCCAACGAACATTAAACGGGTAAAGGTAA
B026	TTTACCCGTTTAATGTTCGTTGGATACTACAAATTAAACTCTAGTA
OSM13	AAGATCCCCAGCTTGTTGATACACCTGCAGGAGGAGAAAGGATCTATGG
OSM14	CGGTACCCGGGGATCCTCTAGAGTCGACCCCTAGGTATAAACGCAGAAAG
OSM35	CAGTTTAGCGGTCTGTTTAAGAGCTATGCTGGAAATAGCAAGTTAAAAT
OSM36	ATTTTAACTTGCTATTTCCAGCATAGCTCTTAAACAGACCGCTAAACTG
OSM37	TAAGAGCTATGCTGGAAACAGCATAGCAAGTTTAAATAAGGCTAGTCCGT
OSM38	ACGGACTAGCCTTATTTAAACTTGCTATGCTGTTTCCAGCATAGCTCTTA

OSM39	GCTTGTTGATACACCTGAGGAGGAGAAAGGATC
OSM40	GATCCTTTCTCCTCCTCAGGTGTATCAACAAGC
OSM47	GCTCAGTCCTAGGTATAATACTAGTGTTTAAGAGCTATGCTGGAAAC
OSM48	GTTTCCAGCATAGCTCTTAAACACTAGTATTATACCTAGGACTGAGC
R65	CATCTGTGCGGTATTTCACACCGCACCTGCAGGCATGCAAGCTTGGCTGT
R66	ACAGCCAAGCTTGCATGCCTGCAGGTGCGGTGTGAAATACCGCACAGATG
R67	GTGCGGTATTTCACACCGCAGTATAAACGCAGAAAGGC
R68	TAGTGTTAACCCCGGGATGGATAAGAAATACTCAATAGG
R69	TCTTATCCATCCCGGGGTTAACACTAGTGTTTAAGAGCTATGCTG
R70	CAAGCTTGCATGCCTGCAGGGGATCCAGTTCACCGACAAAC
R87	GCCTATTGAGTATTTCTTATCCATATCTATATCTCCTTGGATCCTCTAG
R88	CTGTTTCCAGCATAGCTCTTAAACACTAGTGTCGACTCTAGAGGATCCTGTACAACCATAGTGGCCATGAGC
R91	GCCTATTGAGTATTTCTTATCCATATCTATATCTCCTTGGATCCTCTAG
R92	GATGGCTGCCTGAACCATAGTGGCCATGAGC
R93	TGGCCACTATGGTTCAGGCAGCCATCGGAAG
R94	CTGTTTCCAGCATAGCTCTTAAACACTAGTGTCGACTCTAGAGGATCCTGTACATTATACGAGCCGATGATTAATTGTCAAC
R172	TAATCATCGGCTCGTATAATTTCTGTTACTATTAACATATGTTTAAGAGCTATGCTGGAA
R173	TTCCAGCATAGCTCTTAAACATATGTTAATAGTAACAGAAATTATACGAGCCGATGATTA
R176	TAATCATCGGCTCGTATAATGAGTTTAATTTGTAGTATCCGTTTAAGAGCTATGCTGGAA
R177	TTCCAGCATAGCTCTTAAACGGATACTACAAATTAAACTCATTATACGAGCCGATGATTA
115	TGAACTTTAAGAAGGAGATATCATATGGTGAGCAAGGGCGAGGAG

116	AAAACGACGGCCAGTACTAGTTACTTGTACAGCTCGTCCATGCC
117	AGCGACGCCGCAGGGGGATCCGCTCAAGGAAGAGTTCTTCATTGGTC
722	TGATATCTCCTTCTTAAAGTTCAAAGACGACGAGAATTAAGCTTCAAAGAC
723	TGATATCTCCTTCTTAAAGTTCATGTTTGTTCCTTAAAACTACTACCAAACAT
724	TGATATCTCCTTCTTAAAGTTCACCGATGTTGTTGCGGTGCTCG
725	TGATATCTCCTTCTTAAAGTTCAGATCTCATTCTCCGGCGTTCCC
726	TGATATCTCCTTCTTAAAGTTCACATTGCTGCTGGATTACCCAACG
789	AGCGACGCCGCAGGGGGATCCGACGGTTCACAGATCACCACG
790	AGCGACGCCGCAGGGGGATCCCCCGTTAGAGTCGGCAGAAGG
791	AGCGACGCCGCAGGGGGATCCACTAGCACAAAACTACTTTGGTGGTG
792	AGCGACGCCGCAGGGGGATCCGAGCGTCCAATACTCCAAGTGTC
793	AGCGACGCCGCAGGGGGATCCTTTCGTGAAGCGGTAATGAGTTGTGC
848	CAATGAAGAACTCTTCCTTGAGCGGATCGGAATTCTAAAGATCTTTGACAGCTAGC TC
851	AGCGACGCCGCAGGGGGATCCGGATCCAGTTCACCGACAAACAAC
852	AATATTCCTTATCTATATAAGTTTAAGAGCTATGCTGGAAACAGC
853	TTATATAGATAAGGAATATTACTAGTATTATACCTAGGACTGAGCTAG
854	TTCTCACCTTTATATAGATAGTTTAAGAGCTATGCTGGAAACAGC
855	TATCTATATAAAGGTGAGAAACTAGTATTATACCTAGGACTGAGCTAG
856	TAAAGGTAGTCTTAAATGTTGTTTAAGAGCTATGCTGGAAACAGC
857	AACATTTAAGACTACCTTTAACTAGTATTATACCTAGGACTGAGCTAG
858	AAACGCGATAGGCGTGTATGGTTTAAGAGCTATGCTGGAAACAGC
859	CATACACGCCTATCGCGTTTACTAGTATTATACCTAGGACTGAGCTAG
860	TATAACCCATTCTGGATTGCGTTTAAGAGCTATGCTGGAAACAGC
861	GCAATCCAGAATGGGTTATAACTAGTATTATACCTAGGACTGAGCTAG

864 GAGTTTAATTTGTAGTATCCGTTTAAGAGCTATGCTGGAAACAGC

865 GGATACTACAAATTAAACTCACTAGTATTATACCTAGGACTGAGCTAG

866 GTTTATTTATGTGATTTGACGTTTAAGAGCTATGCTGGAAACAGC

867 GTCAAATCACATAAATAAACACTAGTATTATACCTAGGACTGAGCTAG

868 CTTAATTGAGTTTATTTTTAGTTTAAGAGCTATGCTGGAAACAGC

869 TAAAAATAAACTCAATTAAGACTAGTATTATACCTAGGACTGAGCTAG

870 TAATTACTTGATTTAATTGAGTTTAAGAGCTATGCTGGAAACAGC

871 TCAATTAAATCAAGTAATTAACTAGTATTATACCTAGGACTGAGCTAG

872 CTTTCAGCCTTGATAACAAGGTTTAAGAGCTATGCTGGAAACAGC

873 CTTGTTATCAAGGCTGAAAGACTAGTATTATACCTAGGACTGAGCTAG

874 AGCTTGTTTAATTAATTAAAGTTTAAGAGCTATGCTGGAAACAGC

875 TTTAATTAATTAAACAAGCTACTAGTATTATACCTAGGACTGAGCTAG

876 AAAGTACTAATATTACAGAGGTTTAAGAGCTATGCTGGAAACAGC

877 CTCTGTAATATTAGTACTTTACTAGTATTATACCTAGGACTGAGCTAG

878 AGTATTGCATTTGTTTGACAGTTTAAGAGCTATGCTGGAAACAGC

879 TGTCAAACAAATGCAATACTACTAGTATTATACCTAGGACTGAGCTAG

880 AATTCAAAGTTATTTTAGAAGTTTAAGAGCTATGCTGGAAACAGC

881 TTCTAAAATAACTTTGAATTACTAGTATTATACCTAGGACTGAGCTAG

882 ATTCTAAAATAACTTTGAATGTTTAAGAGCTATGCTGGAAACAGC

883 ATTCAAAGTTATTTTAGAATACTAGTATTATACCTAGGACTGAGCTAG

884 ATTAATATTTTATTAACTTAGTTTAAGAGCTATGCTGGAAACAGC

885 TAAGTTAATAAAATATTAATACTAGTATTATACCTAGGACTGAGCTAG

1341 GCGTTTCTACAAACTCTTTTGGGGAATTCTAAAGATCTTTGACAGCTAGCTC

1342 CGTTGTTGCCATTGCTGCAGGTCGAGGATCCAGTTCACCGACAAACAAC

1422 GTTTTTGTTCGGGCCCAAGCTTCTTACTTGTACAGCTCGTCCATGCC

1423	GGCATGGACGAGCTGTACAAGTAAGAAGCTTGGGCCCGAACAAAAAC
1424	GCGTTTCTACAAACTCTTTTGGGGAATTCTAAAGATCTTTGACAGCTAGCTCAGTCCTAG
1492	AGCGACGCCGCAGGGGGATCCTTGACAGCTAGCTCAGTCCTAGGTATAATACTAGTGCTCAAGGAAGAGTTCTTCATTGGTC
1493	AGCGACGCCGCAGGGGGATCCTTTACGGCTAGCTCAGTCCTAGGTATTATACTAGTGCTCAAGGAAGAGTTCTTCATTGGTC
1494	AGCGACGCCGCAGGGGGATCCATAAATGTGAGCGGATAACATTGACATTGTGAGCGGATAACAAGATACTGAGCACGCTCAAGGAAGAGTTCTTCATTGGTC
1496	CAATGAAGAACTCTTCCTTGAGCGGATCTTTACGGCTAGCTCAGTCCTAGGTATTATACTAGTGTTTAAGAGCTATGCTGGAAACAGC
1497	CAATGAAGAACTCTTCCTTGAGCGGATCATAAATGTGAGCGGATAACATTGACATTGTGAGCGGATAACAAGATACTGAGCACGTTTAAGAGCTATGCTGGAAACAGC
1527	ATGATATCTCCTTCTTAAAGTTCATTGACAGCTAGCTCAGTCCTAGG
1659	TTAAAAGTATATTTCAGCAAGTTTAAGAGCTATGCTGGAAACAGC
1660	TTGCTGAAATATACTTTTAAACTAGTATTATACCTAGGACTGAGCTAG
1661	ATACTTAATAAGAATTGTTCGTTTAAGAGCTATGCTGGAAACAGC
1662	GAACAATTCTTATTAAGTATACTAGTATTATACCTAGGACTGAGCTAG
1663	TACTTAATAAGAATTGTTCTGTTTAAGAGCTATGCTGGAAACAGC
1664	AGAACAATTCTTATTAAGTAACTAGTATTATACCTAGGACTGAGCTAG
1665	CCTTTCACACTAATTTGTTCGTTTAAGAGCTATGCTGGAAACAGC
1666	GAACAAATTAGTGTGAAAGGACTAGTATTATACCTAGGACTGAGCTAG
1667	CCCTTTCACACTAATTTGTTGTTTAAGAGCTATGCTGGAAACAGC
1668	AACAAATTAGTGTGAAAGGGACTAGTATTATACCTAGGACTGAGCTAG
1669	CCCGAACAAATTAGTGTGAAGTTTAAGAGCTATGCTGGAAACAGC
1670	TTCACACTAATTTGTTCGGGACTAGTATTATACCTAGGACTGAGCTAG
1671	CCGAACAAATTAGTGTGAAAGTTTAAGAGCTATGCTGGAAACAGC

1672	TTTCACACTAATTTGTTCGGACTAGTATTATACCTAGGACTGAGCTAG
1673	GTGTGAAAGGGTTTGTATCAGTTTAAGAGCTATGCTGGAAACAGC
1674	TGATACAAACCCTTTCACACACTAGTATTATACCTAGGACTGAGCTAG
1675	CATGGCTAATTTTCGAAGCAGTTTAAGAGCTATGCTGGAAACAGC
1676	TGCTTCGAAAATTAGCCATGACTAGTATTATACCTAGGACTGAGCTAG
1711	AGGTTGAGGGGTAACTGGGTGTTTAAGAGCTATGCTGGAAACAGC
1712	ACCCAGTTACCCCTCAACCTACTAGTATTATACCTAGGACTGAGCTAG
1713	CACTACACCCAGTTCTCGAAGTTTAAGAGCTATGCTGGAAACAGC
1714	TTCGAGAACTGGGTGTAGTGACTAGTATTATACCTAGGACTGAGCTAG
1715	GAAAAAGCCTTTCGAGAACTGTTTAAGAGCTATGCTGGAAACAGC
1716	AGTTCTCGAAAGGCTTTTTCACTAGTATTATACCTAGGACTGAGCTAG
1717	GGTGTAGTGATTTCTGTTGCGTTTAAGAGCTATGCTGGAAACAGC
1718	GCAACAGAAATCACTACACCACTAGTATTATACCTAGGACTGAGCTAG
1719	TTCTGTTACTATTAACATATGTTTAAGAGCTATGCTGGAAACAGC
1720	ATATGTTAATAGTAACAGAAACTAGTATTATACCTAGGACTGAGCTAG
1721	AGTTTAATTTGTAGTATCCAGTTTAAGAGCTATGCTGGAAACAGC
1722	TGGATACTACAAATTAAACTACTAGTATTATACCTAGGACTGAGCTAG
1723	AGTATCCAGGGAACATTAAAGTTTAAGAGCTATGCTGGAAACAGC
1724	TTTAATGTTCCCTGGATACTACTAGTATTATACCTAGGACTGAGCTAG
1725	GTATCCAGGGAACATTAAACGTTTAAGAGCTATGCTGGAAACAGC
1726	GTTTAATGTTCCCTGGATACACTAGTATTATACCTAGGACTGAGCTAG
1727	AGGGAACATTAAACGGGTAAGTTTAAGAGCTATGCTGGAAACAGC
1728	TTACCCGTTTAATGTTCCCTACTAGTATTATACCTAGGACTGAGCTAG
1729	CATTAAACGGGTAAAGGTAAGTTTAAGAGCTATGCTGGAAACAGC
1730	TTACCTTTACCCGTTTAATGACTAGTATTATACCTAGGACTGAGCTAG

1731	AGGTAAAGGACAAACGAACAGTTTAAGAGCTATGCTGGAAACAGC
1732	TGTTCGTTTGTCCTTTACCTACTAGTATTATACCTAGGACTGAGCTAG
1733	CAAACGAACATGGCGATTAAGTTTAAGAGCTATGCTGGAAACAGC
1734	TTAATCGCCATGTTCGTTTGACTAGTATTATACCTAGGACTGAGCTAG
1735	CTTCTTAAAGTTCAATTTTTGTTTAAGAGCTATGCTGGAAACAGC
1736	AAAAATTGAACTTTAAGAAGACTAGTATTATACCTAGGACTGAGCTAG
1778	GCGTTTCTACAAACTCTTTTGGTCGATGAGCTGTTTACAATTAATCATCGTGTGG
1779	CGTTGTTGCCATTGCTGCAGGTCGATCTAGACTCCATTTAAATAAAACGAAAGGCT C
1780	GGATCCGAATTTCTACTGTTGTAGATACTAGTAGATCTTTAGAATTCCAGAAATCAT CCTTAG
1781	ATCTACAACAGTAGAAATTCGGATCCATTATACCTAGGACTGAGCTAGCTGTCAAG ATCCTTACTCGAGTCTAGACTGCAGG
1782	CCATTGCTGCAGGTCGATCTAGTTGACAGCTAGCTCAGTCCTAGG
1783	CCATTGCTGCAGGTCGATCTAGTTGACAGCTAGCTCAGTCCTAGG
1796	CTGAGCCTTTCGTTTTATTTAAATGGAGTCTAGTGCAGCCAGCACCAGTCGCCATCT ACAACAGTAGAAATTCGGATC
1797	CTGAGCCTTTCGTTTTATTTAAATGGAGTCTAGTAACTGGGTAGGTCATGAGAATCT ACAACAGTAGAAATTCGGATC
1798	CTGAGCCTTTCGTTTTATTTAAATGGAGTCTAGAATCACTACACCCAGTTCTCATCTA CAACAGTAGAAATTCGGATC
1799	CTGAGCCTTTCGTTTTATTTAAATGGAGTCTAGGTCTACATAAACCTGCAACAATCT ACAACAGTAGAAATTCGGATC
1800	CTGAGCCTTTCGTTTTATTTAAATGGAGTCTAGCCTATATGTTAATAGTAACAATCTA CAACAGTAGAAATTCGGATC
1801	CTGAGCCTTTCGTTTTATTTAAATGGAGTCTAGTTAATGTTCCCTGGATACTAATCTA CAACAGTAGAAATTCGGATC
1802	CTGAGCCTTTCGTTTTATTTAAATGGAGTCTAGTTGTAGTATCCAGGGAACATATCT ACAACAGTAGAAATTCGGATC

1803	CTGAGCCTTTCGTTTTATTTAAATGGAGTCTAGGAACATTAAACGGGTAAAGGATCTACAACAGTAGAAATTCGGATC
1873	GACTCCATTTAAATAAAACGAAAGGCTCAGTCGAAAGACT
1874	CTAGATCGACCTGCAGCAATGGTGCAGGTGCGGTGTGAAATACCG
1875	CTGAGCCTTTCGTTTTATTTAAATGGAGTCTAGGAAATATACTTTTAAATTAAATCTACAACAGTAGAAATTCGGATC
1876	CTGAGCCTTTCGTTTTATTTAAATGGAGTCTAGCCATTGCTGAAATATACTTTATCTACAACAGTAGAAATTCGGATC
1877	CTGAGCCTTTCGTTTTATTTAAATGGAGTCTAGAAAGTATATTTCAGCAATGGATCTACAACAGTAGAAATTCGGATC
1878	CTGAGCCTTTCGTTTTATTTAAATGGAGTCTAGGAGATTCATTTACCATTGCTATCTACAACAGTAGAAATTCGGATC
1880	CTGAGCCTTTCGTTTTATTTAAATGGAGTCTAGACAAATGCAATACTTTTCTTATCTACAACAGTAGAAATTCGGATC
1881	CTGAGCCTTTCGTTTTATTTAAATGGAGTCTAGGTGAAGCATAATCACCTTGTATCTACAACAGTAGAAATTCGGATC
dCas9-M13F	GGTCGATCCCCGCATATAGGTTGTAAAACGACGGCCAGTG
dCas9-TipAR	TACCGAGCTCGAATTCCCCATCTCACCAAGACGCTGGTCG
CRISPR-pSET152-1	TCGCTCCAAGCTGGGCTGTGTGCACGA
CRISPR-pSET152-2	CTCGACCTGGGCGACGCGGCAAATTCCCCAATGTCAAGCAC
FixNco-F3	GCGCGTTGGCCGATTCATTAATG
FixNco-R	GATCGACGGCCTAGGGTTTTAGAGCTAGAAATAG
FixNco-R2	ACATCGTAGCTGACGCCTAC

Table S3B. Oligonucleotides used for sequencing and colony PCR (see Table S2).

**Oligonucleotide**	**Sequence (5´-3´)**

R12	CAGGGACAAGCCACCCGCACA
R13	GGAAGCTAGAGTAAGTAGTTCGC
R71	CGAGTCAGTGAGCGAGGAAG
R72	CAACGTTCAAATCCGCTCCC
R73	ACACGCTTAGAAAATTCACTGATT
R74	TCACCATAGACAAACTCCGATTCA
R75	ACTTTGACCAATTCATCAACAACT
R76	TTGTTTCTTCAGACTTCCGAGT
R77	AGCATCTACTCCACTTGCGT
R78	AGGCTAGTCCGTTATCAACTTGA
R95	TTGTGCCGATAGCTAAGCCT
R96	AAACTTGCTATGCTGTTTCCAG
R169	CGACTCGGTGCCACTTTTTC
R171	CAGCCACGTTTCTGCGAAAA
114	TGATATCTCCTTCTTAAAGTTCAATTTTTCGGCATTGCGCCTTTAATCGC
492	CTCGAACTTCACCTCGGCGC
554	GCGCACCATCTTCTTCAAGG
555	CGGCGGTGATATAGACGTTGTG
766	GACAGCTAGCTCAGTCCTAGGTATAATACTAGT
890	GGAATAAGGGCGACACGGAAATG
891	GAAAATCTTCTCTCATCCGCC
1441	CCCCTGATTCTGTGGATAACCG
1442	GGCAGTTTATGGCGGGCGTC
1491	GCCGTAGAGCGATTGAAGACC
1782	CCATTGCTGCAGGTCGATCTAGTTGACAGCTAGCTCAGTCCTAGG

1787	GAGGTGGCGTTCGCCGCGAGCGATGGACAGGATGTGCACGTC
1788	CTCGGCATGGACGAGCTGTAC
1789	CTGCATGGTGGTGACCACATC
1790	CTCTACTCCCAGCAGATCAACG
1791	GTTCGAGTACGACCTGATCAAGG
1792	GTGCTGCAATGATACCGCGAG
universal M13 Reverse	CAGGAAACAGCTATGAC

Table S3C. Oligonucleotides used for qPCR.

**Oligonucleotide**	**Sequence (5´-3´)**	**Target**
B040	CCCACGTTCACCCCACAAACG	End of CGP3 prophage element
B041	CTAAAATGAAGCCATCGCGACC	End of CGP3 prophage element
B042	ACGTGCTGTTCCTGTGCATGG	*ddh* (NCgl2528, cg2900) control gene
B043	GCTCGGCTAAGACTGCCGCT	*ddh* (NCgl2528, cg2900) control gene

Table S4. Overview of guide RNA spacer sequences.

Spacer name, target region and the spacer sequence of the guide RNAs are listed. Additionally, the orientation of the PAM sequence (5´-spacer-NGG-3´ for sgRNAs, 5´-(T)TTN-spacer-3´ for crRNAs) to the spacer binding position is given.

Spacer	Gene locus of target promoter	Sequence (5´-3´)	Orientation spacer binding position–PAM sequence (5´-3´)
P_{cg1896}_sgRNA-1	cg1896	AATATTCCTTATCTATATAA	5´-spacer-NGG-3´
P_{cg1896}_sgRNA-2	cg1896	TTCTCACCTTTATATAGATA	5´-spacer-NGG-3´
P_{cg1929}_sgRNA-1	cg1929	TAAAGGTAGTCTTAAATGTT	5´-spacer-NGG-3´
P_{cg1940}_sgRNA-1	cg1940	AAACGCGATAGGCGTGTATG	5´-spacer-NGG-3´
P_{cg1940}_sgRNA-2	cg1940	TATAACCCATTCTGGATTGC	5´-spacer-NGG-3´
P_{cg1974}_sgRNA-CS1	cg1974	TTCTGTTACTATTAACATAT	5´-spacer-NGG-3´
P_{cg1974}_sgRNA-CS3	cg1974	GAGTTTAATTTGTAGTATCC	5´-spacer-NGG-3´
P_{cg1974}_sgRNA-25	cg1974	AGGTTGAGGGGTAACTGGGT	5´-spacer-NGG-3´
P_{cg1974}_sgRNA-26	cg1974	CACTACACCCAGTTCTCGAA	5´-spacer-NGG-3´
P_{cg1974}_sgRNA-28	cg1974	GAAAAAGCCTTTCGAGAACT	5´-spacer-NGG-3´
P_{cg1974}_sgRNA-29	cg1974	GGTGTAGTGATTTCTGTTGC	5´-spacer-NGG-3´
P_{cg1974}_sgRNA-33	cg1974	AGTTTAATTTGTAGTATCCA	5´-spacer-NGG-3´
P_{cg1974}_sgRNA-34	cg1974	AGTATCCAGGGAACATTAAA	5´-spacer-NGG-3´
P_{cg1974}_sgRNA-35	cg1974	GTATCCAGGGAACATTAAAC	5´-spacer-NGG-3´
P_{cg1974}_sgRNA-36	cg1974	AGGGAACATTAAACGGGTAA	5´-spacer-NGG-3´
P_{cg1974}_sgRNA-37	cg1974	CATTAAACGGGTAAAGGTAA	5´-spacer-NGG-3´
P_{cg1974}_sgRNA-38	cg1974	AGGTAAAGGACAAACGAACA	5´-spacer-NGG-3´
P_{cg1974}_sgRNA-39	cg1974	CAAACGAACATGGCGATTAA	5´-spacer-NGG-3´
P_{cg1974}_sgRNA-40	cg1974	CTTCTTAAAGTTCAATTTTT	5´-spacer-NGG-3´
P_{cg1974}_crRNA-1	cg1974	ATTTGTAGTATCCAGGGAAC	5´-TTTN-spacer-3´
P_{cg1974}_crRNA-3	cg1974	GGCGACTGGTGCTGGCTGCA	5´-TTTN-spacer-3´
P_{cg1974}_crRNA-4	cg1974	TCTCATGACCTACCCAGTTA	5´-TTTN-spacer-3´

P_{cg1974}_crRNA-5	cg1974	GAGAACTGGGTGTAGTGATT	5´-TTTN-spacer-3´
P_{cg1974}_crRNA-6	cg1974	TGTTGCAGGTTTATGTAGAC	5´-TTTN-spacer-3´
P_{cg1974}_crRNA-7	cg1974	TGTTACTATTAACATATAGG	5´-TTTN-spacer-3´
P_{cg1974}_crRNA-8	cg1974	TAGTATCCAGGGAACATTAA	5´-TTTN-spacer-3´
P_{cg1974}_crRNA-9	cg1974	ATGTTCCCTGGATACTACAA	5´-TTTN-spacer-3´
P_{cg1974}_crRNA-10	cg1974	CCTTTACCCGTTTAATGTTC	5´-TTTN-spacer-3´
P_{cg1977}_sgRNA-1	cg1977	GTTTATTTATGTGATTTGAC	5´-spacer-NGG-3´
P_{cg1999}_sgRNA-1	cg1999	CTTAATTGAGTTTATTTTTA	5´-spacer-NGG-3´
P_{cg1999}_sgRNA-2	cg1999	TAATTACTTGATTTAATTGA	5´-spacer-NGG-3´
P_{cg2004}_sgRNA-1	cg2004	CTTTCAGCCTTGATAACAAG	5´-spacer-NGG-3´
P_{cg2004}_sgRNA-2	cg2004	AGCTTGTTTAATTAATTAAA	5´-spacer-NGG-3´
P_{cg2011}_sgRNA-1	cg2011	AAAGTACTAATATTACAGAG	5´-spacer-NGG-3´
P_{cg2014}_sgRNA-1	cg2014	AGTATTGCATTTGTTTGACA	5´-spacer-NGG-3´
P_{cg2014}_sgRNA-22	cg2014	TTAAAAGTATATTTCAGCAA	5´-spacer-NGG-3´
P_{cg2014}_sgRNA-24	cg2014	ATACTTAATAAGAATTGTTC	5´-spacer-NGG-3´
P_{cg2014}_sgRNA-25	cg2014	TACTTAATAAGAATTGTTCT	5´-spacer-NGG-3´
P_{cg2014}_sgRNA-26	cg2014	CCTTTCACACTAATTTGTTC	5´-spacer-NGG-3´
P_{cg2014}_sgRNA-27	cg2014	CCCTTTCACACTAATTTGTT	5´-spacer-NGG-3´
P_{cg2014}_sgRNA-28	cg2014	CCCGAACAAATTAGTGTGAA	5´-spacer-NGG-3´
P_{cg2014}_sgRNA-29	cg2014	CCGAACAAATTAGTGTGAAA	5´-spacer-NGG-3´
P_{cg2014}_sgRNA-30	cg2014	GTGTGAAAGGGTTTGTATCA	5´-spacer-NGG-3´
P_{cg2014}_sgRNA-31	cg2014	CATGGCTAATTTTCGAAGCA	5´-spacer-NGG-3´
P_{cg2014}_crRNA-1	cg2014	TTAATTTAAAAGTATATTTC	5´-TTTN-spacer-3´
P_{cg2014}_crRNA-2	cg2014	AAAGTATATTTCAGCAATGG	5´-TTTN-spacer-3´
P_{cg2014}_crRNA-3	cg2014	CCATTGCTGAAATATACTTT	5´-TTTN-spacer-3´

P_{cg2014}_crRNA-4	cg2014	AGCAATGGTAAATGAATCTC	5´-TTTN-spacer-3´
P_{cg2014}_crRNA-6	cg2014	AAGAAAAGTATTGCATTTGT	5´-TTTN-spacer-3´
P_{cg2014}_crRNA-7	cg2014	ACAAGGTGATTATGCTTCAC	5´-TTTN-spacer-3´
P_{cg2032}_sgRNA-1	cg2032	AATTCAAAGTTATTTTAGAA	5´-spacer-NGG-3´
P_{cg2032}_sgRNA-2	cg2032	ATTCTAAAATAACTTTGAAT	5´-spacer-NGG-3´
P_{cg2065}_sgRNA-1	cg2065	ATTAATATTTTATTAACTTA	5´-spacer-NGG-3´
$P_{vnz04460}$_sgRNA	*vnz_04460*	AAGGAACCGGACAGTTGAAC	5´-spacer-NGG-3´
*vnz04465*_sgRNA	*vnz_04465*	TTTCATGACGACCCCGGACG	5´-spacer-NGG-3´

FIGURES SUPPLEMENTARY MATERIAL

A

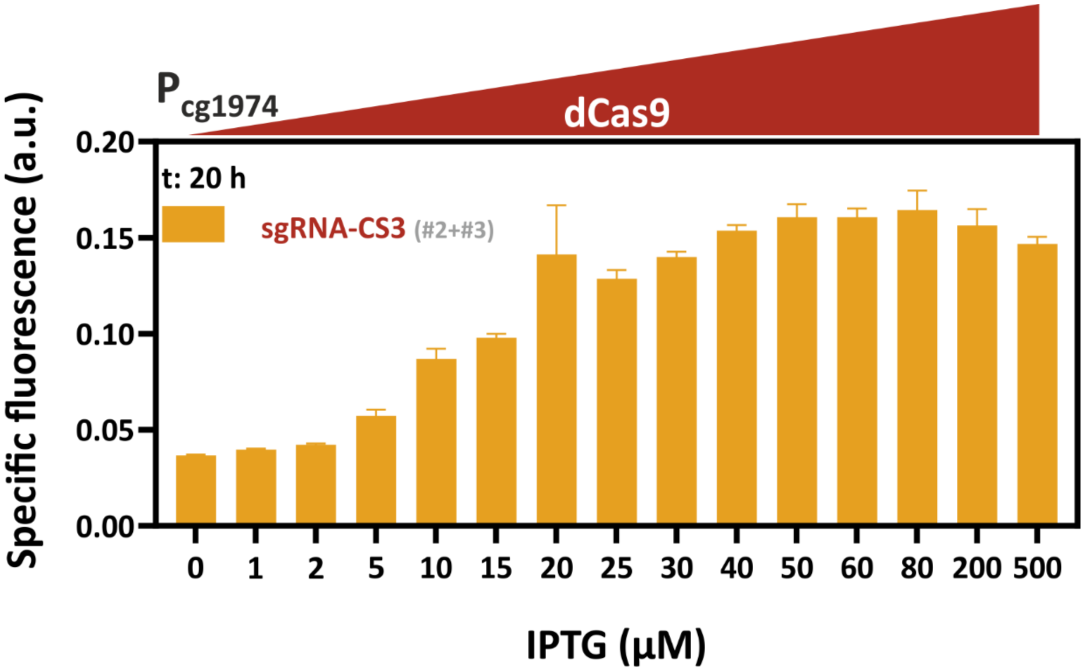

B

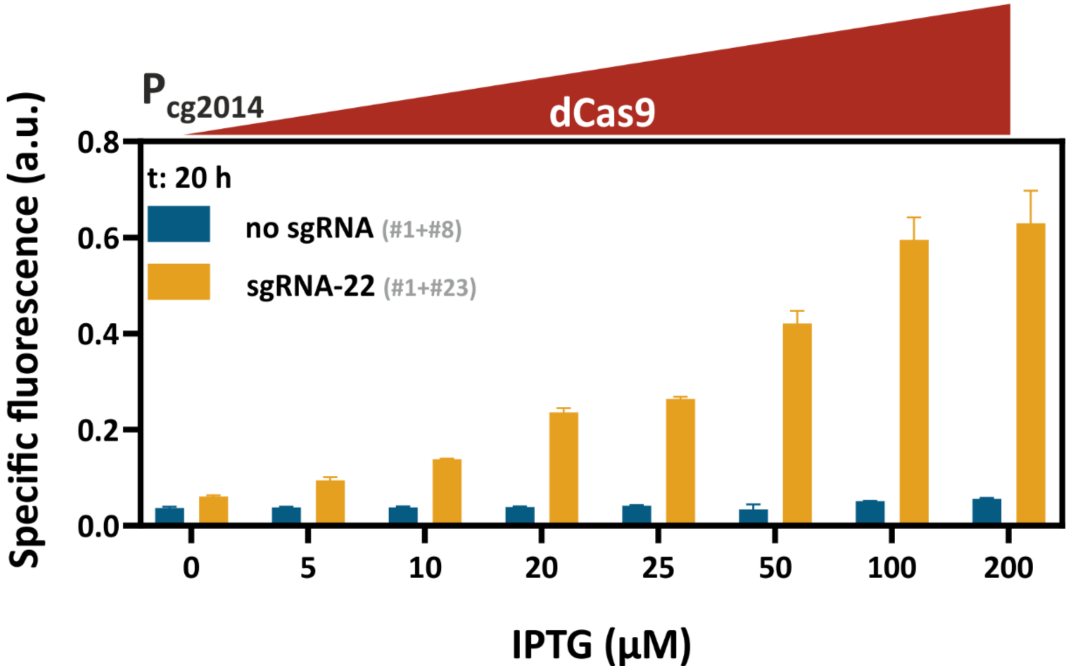

Figure S1. Correlation of CRISPRcosi efficiencies at prophage promoter P_{cg1974} (A) and P_{cg2014} (B) and *dcas9* expression levels. Systematic dose-response analysis with increasing amounts of IPTG to induce LacI/P_{tac}-controlled *dcas9* expression. Bar plots

represent backscatter-normalized reporter outputs (t: 20 h; specific Venus fluorescence) of prophage-free, *cgpS* expressing *C. glutamicum* strains (Δphage::P_{cgpS}-*cgpS*, (5)) that were cultivated in CGXII minimal medium supplemented with 111 mM glucose, 25 µg/mL kanamycin, 10 µg/mL chloramphenicol and varying IPTG concentrations in the BioLector I® microtiter cultivation system (n=3). (A) Cells harboured plasmids #3 and #2, which provided the P_{cg1974}-*venus* reporter system and allowed for constitutive cg1974_sgRNA-CS3 as well as IPTG-inducible *dcas9* expression. (B) Cells harboured plasmid #1 allowing for IPTG-inducible *dcas9* expression and plasmid #23 or #8, which provided the P_{cg2014}-*venus* reporter system with or without cg2014_sgRNA-22, respectively. For plasmid IDs (#number), refer to Table S2.

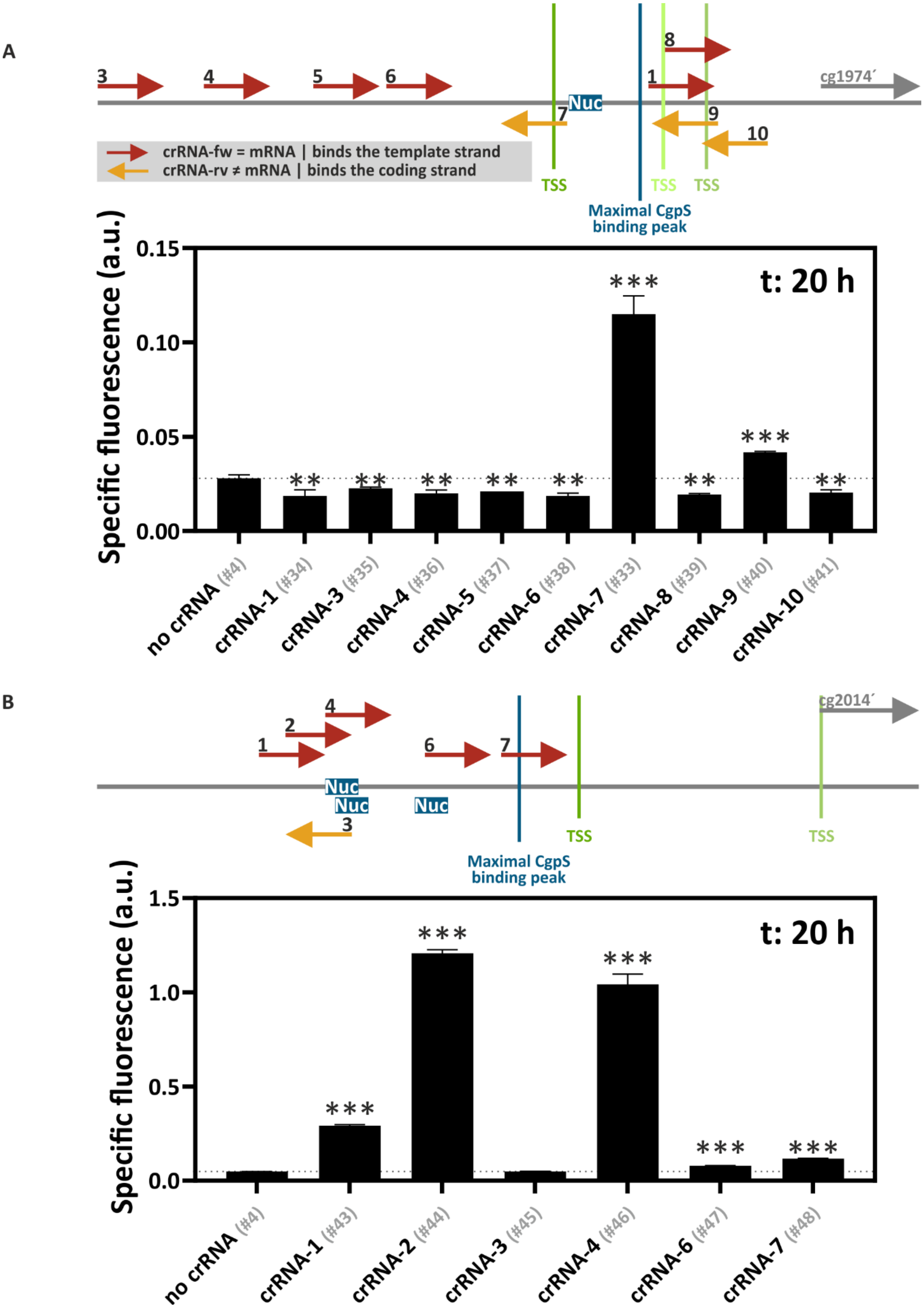
A
3
4
5
6
7
8
1
9
10
Nuc
cg1974´
crRNA-fw = mRNA | binds the template strand
crRNA-rv ≠ mRNA | binds the coding strand
TSS
TSS
TSS
Maximal CgpS binding peak
Specific fluorescence (a.u.)
0.15
0.10
0.05
0.00
t: 20 h
***
***
**
no crRNA (#4)
crRNA-1 (#34)
crRNA-3 (#35)
crRNA-4 (#36)
crRNA-5 (#37)
crRNA-6 (#38)
crRNA-7 (#33)
crRNA-8 (#39)
crRNA-9 (#40)
crRNA-10 (#41)
B
1
2
3
4
6
7
Nuc
Nuc
Nuc
cg2014´
TSS
TSS
Maximal CgpS binding peak
Specific fluorescence (a.u.)
1.5
1.0
0.5
0.0
t: 20 h
***
no crRNA (#4)
crRNA-1 (#43)
crRNA-2 (#44)
crRNA-3 (#45)
crRNA-4 (#46)
crRNA-6 (#47)
crRNA-7 (#48)

Figure S2. The crRNA binding positions determine the dCas12a-mediated CRISPRcosi efficiency. Arrows in the graphical representations show the target position and DNA strand of the different crRNAs designed for dCas12a guiding to P_{cg1974} (A) and P_{cg2014} (B). The first 30 nucleotides of the corresponding cg1974 and cg2014 genes are shown as grey arrows and the positions of maximal CgpS binding are given as blue vertical lines, while the vertical lines in shades of green indicate the positions of the previously identified TSS positions (TSS colors were mapped according to the ranking of their enrichment scores: dark green > light green) (5). Bar plots represent backscatter-normalized reporter outputs (t: 20 h; specific Venus fluorescence) of prophage-free, *cgpS* expressing *C. glutamicum* strains (Δphage::P_{cgpS}-*cgpS*, (5)) harbouring one of the different pEC plasmids containing no (#4) or one of the crRNA encoding sequences ((A): P_{cg1974}_crRNA-1 (#34), -3(#35), -4 (#36), -5, (#37), -6 (#38), -7 (#33), -8 (#39), -9 (#40), -10 (#41); (B): P_{cg2014}_crRNA-1 (#43),-2 (#44),-3 (#45),-4(#46),-6 (#47), -7 (#48)) in combination with the pJC1-based plasmids #32 (A) or #42 (B) that provided the phage promoter-*venus* reporter system and allowed for the constitutive *dcas12a* expression. The reporter levels of cells lacking any crRNA are given as dotted grey horizontal lines. Number of asterisks above bars indicate the significance levels of crRNA effects (unpaired t test, ***two-tailed *p*-value<0.005, **two-tailed *p*-value<0.05). Strains were cultivated in the BioLector I® microtiter cultivation system in CGXII minimal medium containing 111 mM glucose, 25

µg/mL kanamycin and 10 µg/mL chloramphenicol (n=3). For plasmid IDs (#number), refer to Table S2.

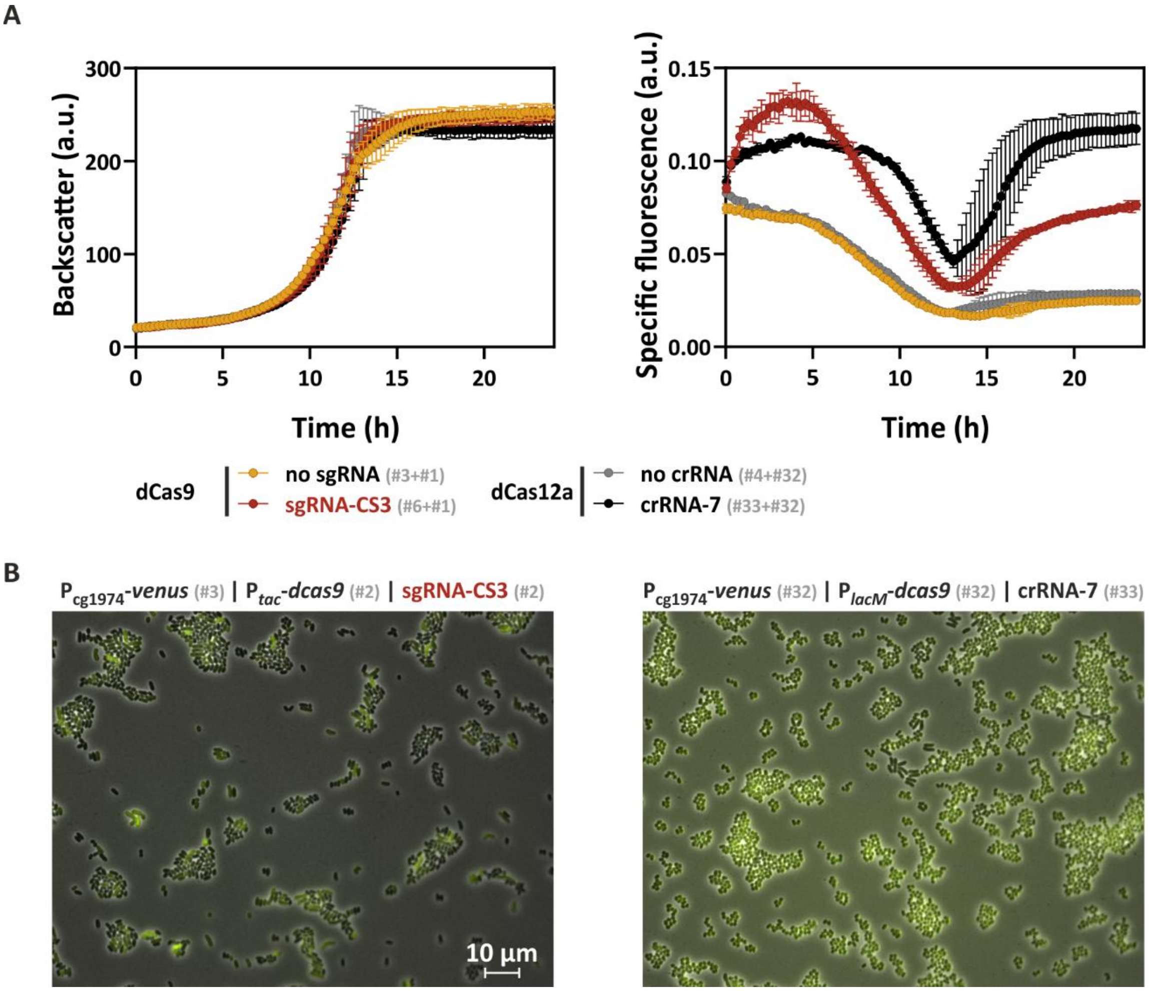

Figure S3. Supplementary information on Figure 5: dCas9 and dCas12a both mediate efficient CRISPRcosi. (A) The graphs represent the backscatter values and the time-resolved, backscatter-normalized reporter outputs (specific Venus fluorescence) of prophage-free, *cgpS* expressing *C. glutamicum* strains (Δphage::P$_{cgpS}$-*cgpS*, (5)) harbouring

the P_{cg1974}-*venus* reporter construct and either the *dcas9* gene combined with the sgRNA-CS3-encoding sequence (#6+#1) or the *dcas12a* gene in concert with the crRNA-7 encoding sequence (#33+#32). Plasmid combinations lacking any guide RNAs encoding sequences (dCas9: #3+#1; dCas12a: #4+#32) served as controls. Strains were cultivated in the BioLector I® microtiter cultivation system in CGXII minimal medium containing 111 mM glucose, 25 µg/mL kanamycin, 10 µg/mL chloramphenicol and 200 µM IPTG (n=3). The drop in specific Venus fluorescence, which started after about five h of cultivation, is characteristic of fast-growing *C. glutamicum* cells and is caused by oxygen limitation leading to delayed oxygen-dependent maturation of the Venus protein. After entering the stationary phase, dissolved oxygen availability increased, and fluorescence signals normalized. (B) Representative fluorescence microscopy images of prophage-free, *cgpS* expressing *C. glutamicum* strains (Δphage::P_{cgpS}-*cgpS*, (5)) harbouring the P_{cg1974}-*venus* reporter construct and either the *dcas9* gene combined with the sgRNA-CS3-encoding sequence (#3+#2) or the *dcas12a* gene in concert with the crRNA-7 encoding sequence (#32+#33). The scale bar represents 10 µm. For plasmid IDs (#number), refer to Table S2.

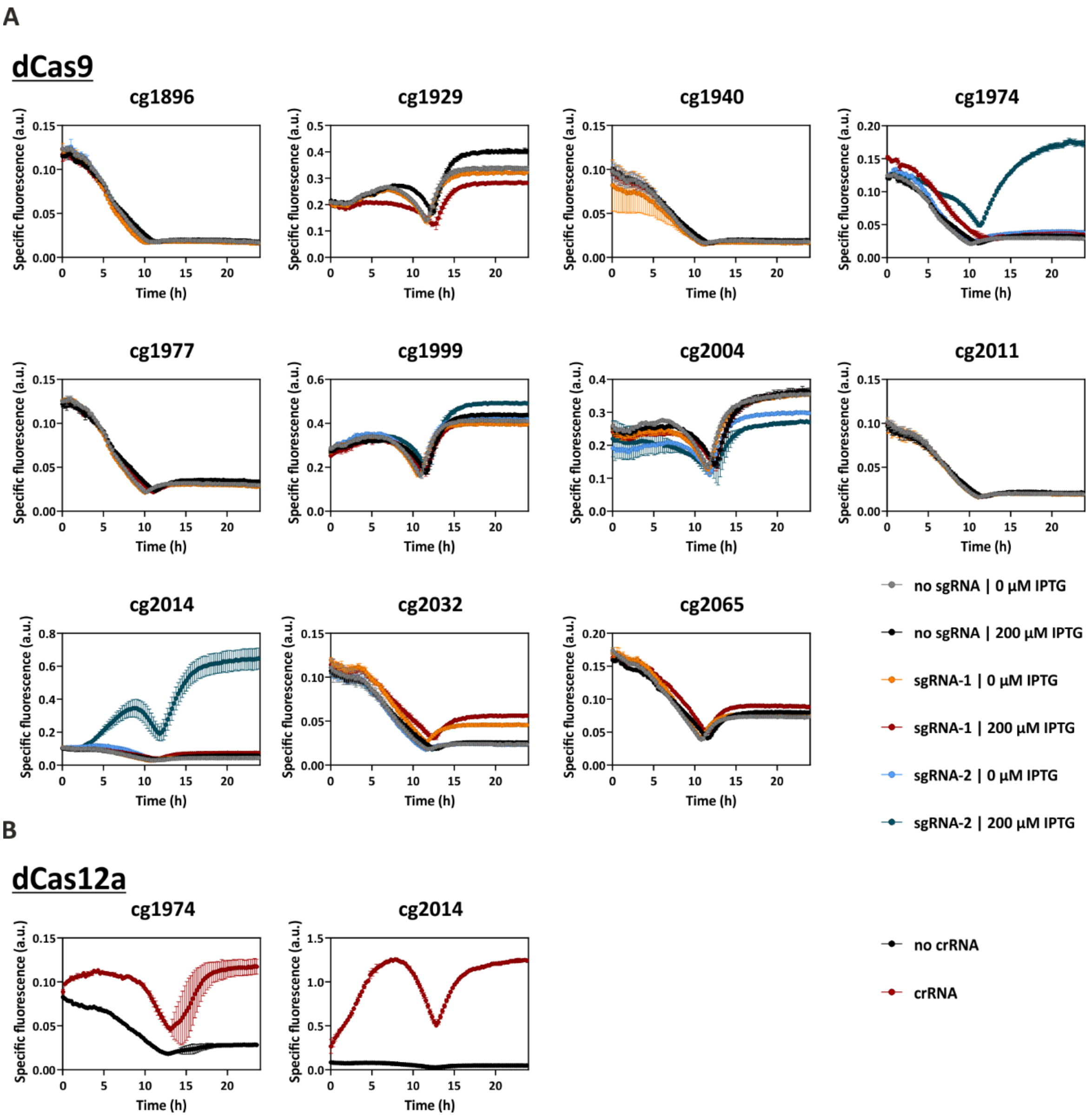

Figure S4. Supplementary information on Figure 6: CRISPRcosi counteracts CgpS-mediated silencing at different prophage promoters. Time-resolved representation of the backscatter-normalized specific Venus fluorescence values of all different strains shown in Figure 6. Graphs represent backscatter-normalized reporter outputs (specific Venus fluorescence) in the course of cultivation of cells with and without guide RNA

encoding sequences. (A) Prophage-free, *cgpS* and *dcas9* co-expressing *C. glutamicum* cells (Δphage::P_{cgpS}-*cgpS* (5)) harbouring the *dcas9* encoding, sgRNA-free plasmid #1 served as platform strain. Guide RNA-free strains were equipped with pJC1-plasmids containing the sequences of the different prophage promoters fused to the reporter gene *venus* to monitor promoter activities (P_{cg1896}: #49; P_{cg1929}: #50; P_{cg1940}: #51; P_{cg1974}: #3; P_{cg1977}: #52; P_{cg1999}: #53; P_{cg2004}: #54; P_{cg2011}: #55; P_{cg2014}: #8; P_{cg2032}: #56; P_{cg2065}: #57). P_{J23319}-mediated expression of specific guide RNAs was achieved by inserting the sgRNA encoding sequences into the pJC1-plasmids (P_{cg1896}: sgRNA-1 (#58) and -2 (#59); P_{cg1929}: sgRNA-1 (#60); P_{cg1940}: sgRNA-1 (#61) and -2 (#62); P_{cg1974}: sgRNA-CS1 (#13) and -CS3 (#6); P_{cg1977}: sgRNA-1 (#64); P_{cg1999}: sgRNA-1 (#65) and -2 (#66); P_{cg2004}: sgRNA-1 (#67) and -2 (#68); P_{cg2011}: sgRNA-1 (#69); P_{cg2014}: sgRNA-1 (#70) and -22 (#23), P_{cg2032}: sgRNA-1 (#71) and -2 (#72); P_{cg2065}: sgRNA-1 (#73)). (B) Prophage-free, *cgpS* expressing *C. glutamicum* cells (Δphage::P_{cgpS}-*cgpS* (5)) harboured the pJC1-based plasmids containing the *dcas12a* gene and the phage promoter fused to the reporter gene *venus*. crRNA-free plasmid #4 served as control, while plasmids #33 ($P_{cg1974_}$crRNA-7) and #44 ($P_{cg2014_}$crRNA-2) were used for P_{J23319}-mediated expression of specific crRNAs. The drop in specific Venus fluorescence is caused by a delayed oxygen-dependent maturation of the Venus protein during exponential growth. Fluorescence signals normalize after cells have entered the stationary phase and the levels of dissolved oxygen increased. This effect does not affect the 20 h measurement provided in the main text, which allows for a fair

comparison. Strains were cultivated in the BioLector I® microtiter cultivation system in CGXII minimal medium containing 111 mM glucose, 25 µg/mL kanamycin, 10 µg/mL chloramphenicol and 0 or 200 µM IPTG (n=3). For plasmid IDs (#number), refer to Table S2.

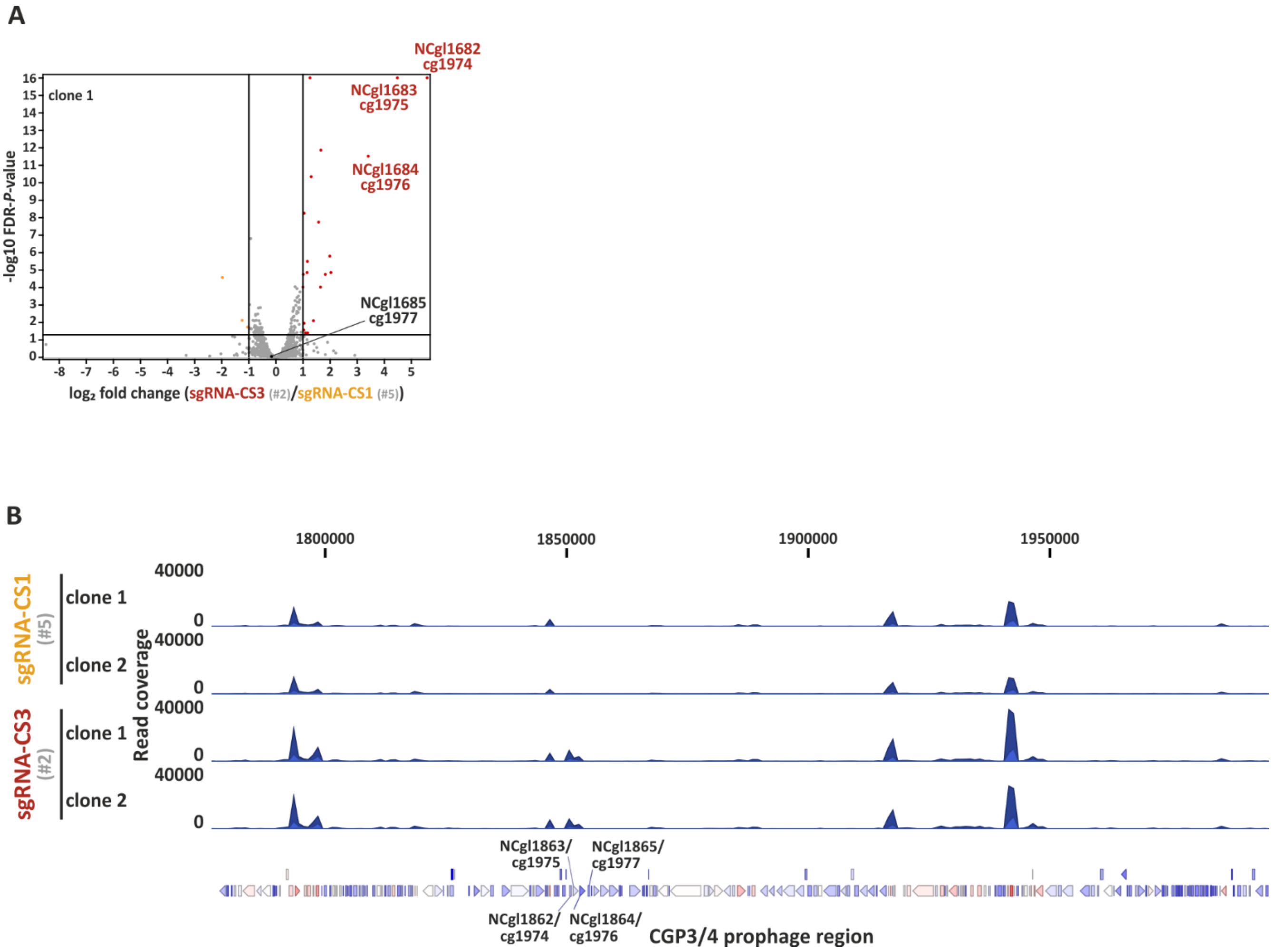

Figure S5. Supplementary data on Figure 7: Investigation of potential off-target effects of CRISPRcosi. Analysis of gene expression levels of *C. glutamicum* wild-type cells

harbouring the pEC-based plasmids that allowed for IPTG-inducible *dcas9* expression and constitutive expression of sgRNA-CS3 (#2) in comparison to control cells expressing sgRNA-CS1 (#5). Both strains were cultivated in duplicates in CGXII minimal medium supplemented with 111 mM glucose, 10 µg/mL chloramphenicol and 200 µm IPTG for 7 h. (A) Red and orange dots in the volcano plot represent significantly up- and downregulated genes (FDR-*P*-values<0.05 and $|\log_2$ fold change$|>1$), respectively, in cells expressing sgRNA-CS3 (clone 1) in comparison to cells expressing sgRNA-CS-1 (clone 1+2). Non-significant hits are given as grey dots (FDR-*P*-values>0.05 or $|\log_2$ fold change$|<1$), while the corresponding significance thresholds are represented by black lines. (B) RNA-seq read coverage profiles plotted over the CGP3/4 prophage region of clones expressing sgRNA-CS1 and sgRNA-CS3. Coverage data aggregation was set to over 100 bp. Prophage genes are shown as arrows below the plots, highlighted are genes cg1974-77 (NCgl1682-85). For plasmid IDs (#number), refer to Table S2.

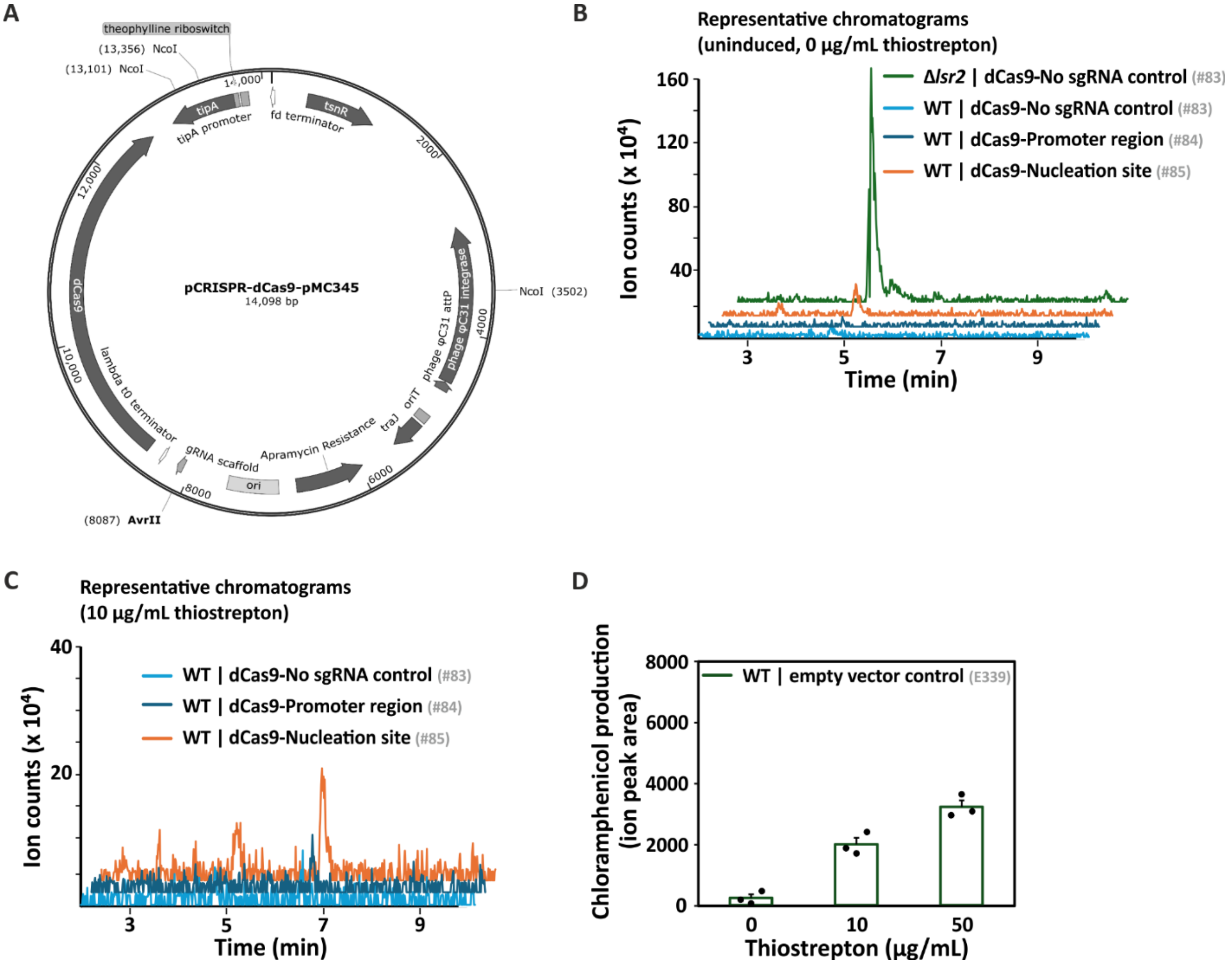

Figure S6. Supplementary data on Figure 9: Application of CRISPRcosi to increase antibiotic production by *Streptomyces*. (A) Map of an integrative and inducible dCas9 plasmid, derived from pCRISPR-dCas9 (Addgene plasmid #125687, #AT, (15)). The original NcoI sgRNA cloning site was altered to generate a unique AvrII site. (B) Representative ion chromatograms of chloramphenicol ($[M-H]^-$ at *m/z* 321.00). The input samples were metabolites extracted from non-induced (0 µg/mL thiostrepton) *S. venezuelae* wild-type (WT) carrying empty dCas9 plasmid control (no sgRNA, light blue, #83), sgRNA-vnz04460 (promoter-targeting, dark blue, #84), sgRNA-vnz04465 (nucleation site-targeting, orange,

#85), or a Δ*lsr2* mutant carrying the empty plasmid as a positive uninduced control (green, #83). (C) Similar to (B) except the samples were from the same dCas9 construct-carrying *S. venezuelae* wild-type strains grown under thiostrepton induction (10 μg/mL). Chloramphenicol was eluted at 4.75 min under the gradient used in the mobile phase, shown by major peaks close to the 5 min mark in the figures. (D) Bar plots represent chloramphenicol production by wild-type *S. venezuelae* carrying empty plasmid control E339 (pIJ6902-hyg: no dCas9, no sgRNA, thiostrepton resistant). Thiostrepton (10 or 50 μg/mL) was added to the culture, after which chloramphenicol production was assessed, as described for Fig. 9. Error bars indicate standard error of three biological replicates of MYM liquid cultures grown for 3 days. The value of each replicate is depicted as a black filled circle. These results showed that thiostrepton stimulated the chloramphenicol production in *S. venezuelae*, but not to the same extend as observed with CRISPRcosi (Figure 9B). For plasmid IDs (#number), refer to Table S2.

REFERENCES

1. MacNeil DJ, Gewain KM, Ruby CL, Dezeny G, Gibbons PH, MacNeil T. 1992. Analysis of *Streptomyces avermitilis* genes required for avermectin biosynthesis utilizing a novel integration vector. Gene 111:61-8.
2. Kinoshita S, Udaka S, Shimono M. 1957. Studies on the amino acid fermentation - Part I. Production of L-glutamic acid by various microorganisms. J Gen Appl Microbiol 3:193-205.
3. Helfrich S, Pfeifer E, Krämer C, Sachs CC, Wiechert W, Kohlheyer D, Nöh K, Frunzke J. 2015. Live cell imaging of SOS and prophage dynamics in isogenic bacterial populations. Mol Microbiol 98:636-650.
4. Baumgart M, Unthan S, Rückert C, Sivalingam J, Grünberger A, Kalinowski J, Bott M, Noack S, Frunzke J. 2013. Construction of a prophage-free variant of *Corynebacterium glutamicum* ATCC 13032 for use as a platform strain for basic research and industrial biotechnology. Appl Environ Microbiol 79:6006-15.
5. Wiechert J, Filipchyk A, Hünnefeld M, Gätgens C, Brehm J, Heermann R, Frunzke J. 2020. Deciphering the rules underlying xenogeneic silencing and counter-silencing of Lsr2-like proteins using CgpS of *Corynebacterium glutamicum* as a model. mBio 11.
6. Gomez-Escribano JP, Holmes NA, Schlimpert S, Bibb MJ, Chandra G, Wilkinson B, Buttner MJ, Bibb MJ. 2021. *Streptomyces venezuelae* NRRL B-65442: genome sequence of a model strain used to study morphological differentiation in filamentous actinobacteria. J Ind Microbiol Biotechnol 48.
7. Gehrke EJ, Zhang X, Pimentel-Elardo SM, Johnson AR, Rees CA, Jones SE, Hindra, Gehrke SS, Turvey S, Boursalie S, Hill JE, Carlson EE, Nodwell JR, Elliot MA. 2019. Silencing cryptic specialized metabolism in Streptomyces by the nucleoid-associated protein Lsr2. eLife 8:e47691.
8. Tong Y, Charusanti P, Zhang L, Weber T, Lee SY. 2015. CRISPR-Cas9 Based Engineering of Actinomycetal Genomes. ACS Synth Biol 4:1020-9.
9. Eikmanns BJ, Thum-Schmitz N, Eggeling L, Lüdtke KU, Sahm H. 1994. Nucleotide sequence, expression and transcriptional analysis of the Corynebacterium glutamicum gltA gene encoding citrate synthase. Microbiology (Reading) 140 (Pt 8):1817-28.
10. Pfeifer E, Hünnefeld M, Popa O, Polen T, Kohlheyer D, Baumgart M, Frunzke J. 2016. Silencing of cryptic prophages in *Corynebacterium glutamicum*. Nucleic Acids Res 44:10117-10131.
11. Kirchner O, Tauch A. 2003. Tools for genetic engineering in the amino acid-producing bacterium *Corynebacterium glutamicum*. J Biotechnol 104:287-299.
12. Bakkes PJ, Ramp P, Bida A, Dohmen-Olma D, Bott M, Freudl R. 2020. Improved pEKEx2-derived expression vectors for tightly controlled production of recombinant proteins in *Corynebacterium glutamicum*. Plasmid 112:102540.
13. Chen B, Gilbert LA, Cimini BA, Schnitzbauer J, Zhang W, Li GW, Park J, Blackburn EH, Weissman JS, Qi LS, Huang B. 2013. Dynamic imaging of genomic loci in living human cells by an optimized CRISPR/Cas system. Cell 155:1479-91.
14. Lausberg F, Chattopadhyay AR, Heyer A, Eggeling L, Freudl R. 2012. A tetracycline inducible expression vector for *Corynebacterium glutamicum* allowing tightly regulable gene expression. Plasmid 68:142-147.
15. Tong Y, Charusanti P, Zhang L, Weber T, Lee SY. 2015. CRISPR-Cas9 based engineering of actinomycetal genomes. ACS Synth Biol 4:1020-9.
16. Bierman M, Logan R, O'Brien K, Seno ET, Rao RN, Schoner BE. 1992. Plasmid cloning vectors for the conjugal transfer of DNA from *Escherichia coli* to *Streptomyces* spp. Gene 116:43-9.

17. Baumgart M, Luder K, Grover S, Gätgens C, Besra GS, Frunzke J. 2013. IpsA, a novel LacI-type regulator, is required for inositol-derived lipid formation in *Corynebacteria* and *Mycobacteria*. BMC Biol 11:122.
